# A neuronal GPCR modifies neuropeptide signaling to suppress proteostasis in distal tissues

**DOI:** 10.1126/sciadv.ady6048

**Published:** 2026-10-09

**Authors:** Joud Hirbawi, Adam Zaretsky, Huadong Zhu, Uri Goshtchevsky, Gourab Dey, Emmanuelle Merquiol, Atif Ahmed Siddiqui, Irit Cohen, Galia Blum, Ehud Cohen

**Affiliations:** ^1^ Department of Biochemistry and Molecular Biology, the Institute for Medical Research Israel–Canada, The Hebrew University of Jerusalem, Jerusalem, Israel.; ^2^ Institute for Drug Research, School of Pharmacy, Faculty of Medicine, The Hebrew University, Jerusalem, Israel.

## Abstract

The proteostasis network (PN) safeguards the integrity of proteins by promoting various cellular activities. However, with aging, the PN’s competence declines and aggregated proteins accrue within cells. This process underlies the development of neurodegenerative disorders such as Alzheimer’s and Huntington’s diseases. While the PN functions within cells, proteostasis is regulated across the organism by intertissue communication, which is partially governed by neurons. We previously found that reducing the expression of *gtr-1*, which encodes a neuronal heterotrimeric guanine nucleotide–binding protein–coupled receptor, mitigates the toxicity of the Alzheimer’s disease cause, Aβ peptide. Here, we investigated the mechanism that is acted upon the knockdown of *gtr-1* and found that it differentially modulates gene expression profiles, including of neuropeptide-coding genes, and dissimilarly modifies protein aggregation in worms that express distinct proteotoxic proteins. The knockdown of *gtr-1* also enhances protein degradation. These findings highlight the roles of neuropeptides as organismal coordinators of proteostasis in the face of distinct proteotoxic challenges.

## INTRODUCTION

Protein maturation is a complex, multistep process that requires the assistance and supervision of specialized mechanisms that help newly synthesized polypeptides attain their desired folding states and form proper interactions with their molecular partners. Nonetheless, not all nascent proteins maturate properly, and subsets of aggregation-prone polypeptides terminally misfold. These molecules, as well as damaged mature proteins, are directed for degradation by the ubiquitin proteasome system (UPS) or by autophagy. Early in life, these coordinated protein quality control activities efficiently ensure protein homeostasis (proteostasis); however, in late stages of life, the competence of these mechanisms declines (*1*), and aggregated proteins escape the cellular surveillance and accumulate within cells (*2*). This process jeopardizes proteostasis (*3*) and can lead to the development of various disorders that are collectively known as “proteinopathies” (*4*). Neurodegenerative diseases such as Alzheimer’s disease (AD) (*5*) and Huntington’s disease (HD) (*6*) are incurable, late-onset proteinopathies that stem from toxic protein aggregation (proteotoxicity) (*7*).

AD develops as a result of the accumulation and aggregation of peptides that are produced by dual proteolytic digestion of the type 1 transmembrane protein, amyloid precursor protein (*8*). These peptides, which are known as “Amyloid β (Aβ) peptides,” form oligomers and large aggregates that accumulate in the brain. Oligomers are the most toxic Aβ species that probably underlie the manifestation of AD (*9*). Similarly, abnormally long poly-glutamine (polyQ) expansions in the sequence of certain proteins render them prone to aggregate and trigger the development of several incurable neurodegenerative maladies including HD and different types of ataxia (*6*).

The late-onset feature, common to various neurodegenerative illnesses, raised the prospect that aging exposes the older organism to these maladies by suppressing the activities of proteostasis-promoting mechanisms. This theme has been supported by the discovery that the alteration of aging alleviates proteotoxicity of neurodegeneration-causing proteins in model nematodes (*Caenorhabditis elegans*) and in mice. For instance, reducing the activity of the insulin/insulin-like growth factor (IGF) signaling cascade (IIS), a prominent aging-regulating pathway (*10*), by the knockdown of the nematode’s sole IIS receptor *daf-2*, protects worms that express Aβ in their muscles from paralysis that stems from the aggregation of this peptide (*11*). Similarly, IIS reduction mitigates the toxicity of abnormally long polyQ stretches (*12*) and of additional neurodegeneration-causing proteins (*13*, *14*). This protective mechanism is conserved from worms to mammals, as AD-model mice that exhibit reduced IGF1 signaling are protected from Aβ-promoted proteotoxicity (*15*, *16*). In nematodes, these protective effects are entirely dependent on the activities of a set of aging-controlling transcription factors, including DAF-16/FOXO, SKN-1/NRF and the heat shock factor 1 (HSF-1), which were reported to regulate proteostasis (*11*, *17*). The zinc finger transcription factor PQM-1 is also involved in the regulation of proteostasis, though it appears to play opposing roles in the face of distinct proteotoxic proteins. While it was shown to mitigate the toxicity of polyQ stretches (*18*), the knockdown of *pqm-1* protects worms from Aβ-mediated toxicity (*19*).

Since the proteostasis network encompasses mechanisms that act within cells, it was thought that proteostasis is regulated cell-autonomously. Nevertheless, proteostasis was found to be also regulated at the organismal level by intertissue communication (*20*). Neurons regulate both the activation of the heat shock response (HSR) in remote tissues of worms (*21*) and the rates of proteotoxicity in nematodes that express aggregative proteins in their muscles (*22*). We previously found that the heterotrimeric guanine nucleotide–binding protein–coupled receptor (GPCR) thermal receptor 1 (GTR-1), which appears to be expressed primarily in neurons, governs HSR activation and Aβ-mediated proteotoxicity in opposing manners. While the knockdown of *gtr-1* by RNA interference (RNAi) reduces the survival rates of heat-stressed worms, it mitigates Aβ-mediated proteotoxicity in the worms’ muscles (*23*). This finding raised the query of what mechanisms downstream of GTR-1 orchestrate proteostasis across tissues.

Using proteotoxicity model nematodes, we found that the enhancement of proteostasis that stems from the knockdown of *gtr-1*, is not Aβ specific but also protects animals that express the aggregation-prone polyQ35-YFP (yellow fluorescent protein) in their muscles. While the counter Aβ proteotoxic effect of *gtr-1* RNAi is dependent on the transcription factor SKN-1, the mitigation of polyQ35-YFP requires DAF-16. Comparing gene expression modulations in Aβ- and polyQ35-YFP–expressing worms, we characterized distinct transcriptomic modulations primarily in genes that encode proteases and a subgroup of neuropeptides. Physiological assays unveiled that these neuropeptides are needed for the counter-proteotoxic effect of *gtr-1* RNAi treatment. Yet, *nlp-59*, one of these neuropeptides, was found to be vital for protection from polyQ35-YFP but dispensable for the mitigation of Aβ-promoted proteotoxicity. We also found that the knockdown of *gtr-1* opposingly affects the aggregation rates of Aβ and of polyQ35-YFP, whereas Aβ aggregation is reduced upon treatment with *gtr-1* RNAi; this treatment augments the aggregation of polyQ35-YFP. Furthermore, *gtr-1* RNAi enhances protein degradation by elevating the activities of both UPS and autophagy. Our results uncover a mechanism that coordinates proteostasis at the organismal level and raise the prospect that the infusion of specifically tailored combination of neuropeptides, and perhaps of other signaling molecules, could be useful to postpone, and perhaps even prevent, the manifestation of late-onset neurodegenerative maladies.

## RESULTS

### The knockdown of *gtr-1* mitigates polyQ-mediated proteotoxicity by enhancing aggregation

Since components of the proteostasis network could play opposing roles in the face of distinct proteotoxic challenges (*24*), we asked whether the knockdown of *gtr-1*, which alleviates Aβ-mediated proteotoxicity when knocked down throughout the worm (*23*), or primarily in neurons (fig. S1A), is also capable of mitigating proteotoxicity that emanates from the expression of polyQ stretches. To address this, we used worms that express a chimeric, aggregation-prone protein that is composed of 35 glutamine repeats, fused to the YFP (polyQ35-YFP), in their body wall muscles, (strain AM140). This expression results in motility impairments (*12*) that can be measured by the thrashing assay (*25*). AM140 animals were treated from hatching with RNAi that targets either *daf-2* or the *gtr-1* 3′ untranslated region [hereafter, *gtr-1* RNAi. This RNAi efficiently reduces the expression of GTR-1 (fig. S1B)]. An additional group of worms were grown on bacteria that harbor the empty vector (EV) and served as control. Using the thrashing assay, we found that while the knockdown of *daf-2* resulted in elevated thrashing rates in both day 4 and day 6 of adulthood, *gtr-1* RNAi–treated animals exhibited elevated thrashing rates at day 6 of adulthood but not at day 4 (Fig. 1A).

**Fig. 1.**
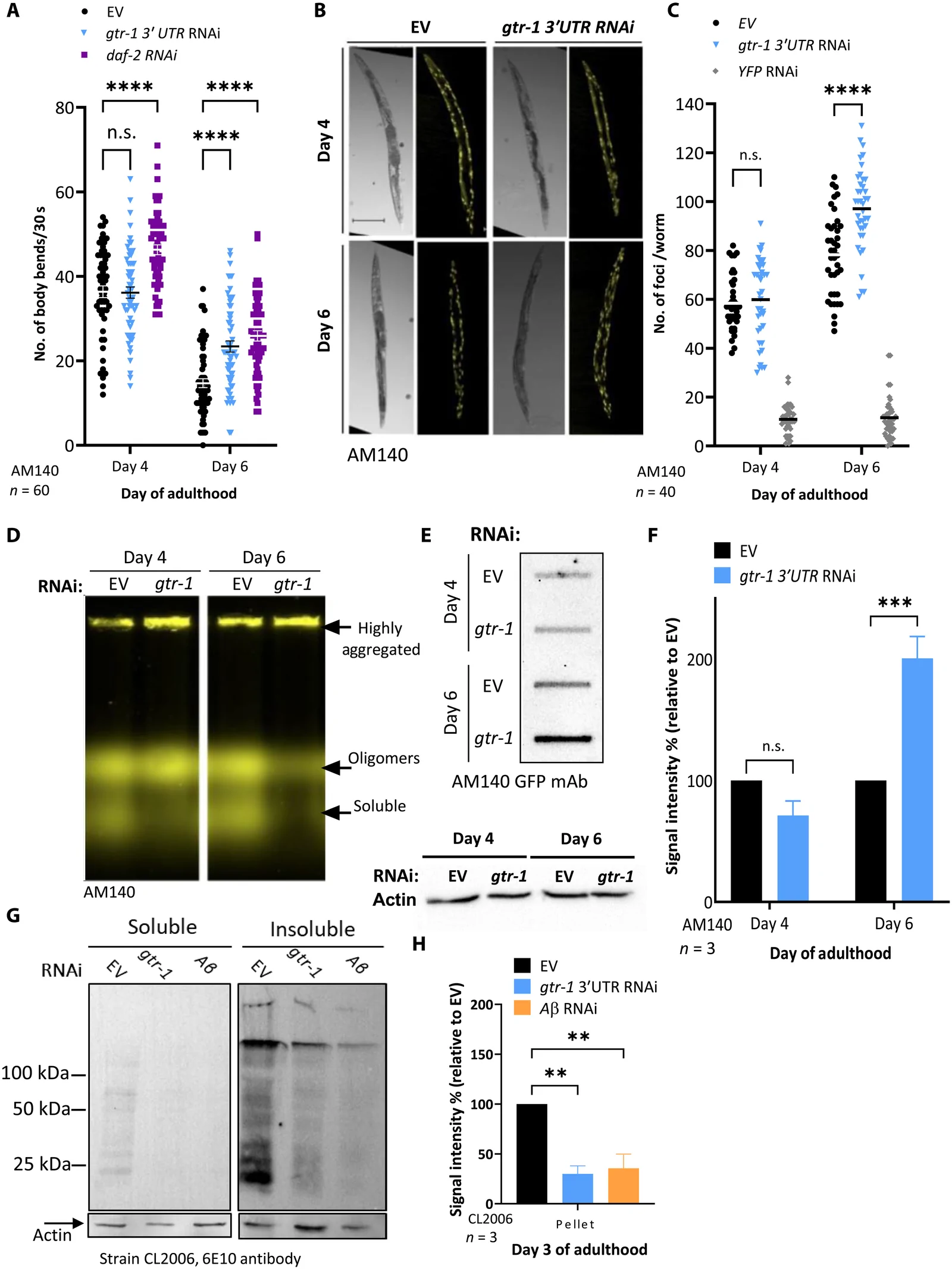
The knockdown of *gtr-1* mitigates polyQ35-YFP toxicity by promoting hyperaggregation. (**A**) Six-day-old, *gtr-1* RNAi–treated AM140 worms show higher thrashing rate compared with their age-matched untreated counterparts. This effect was similar to that of *daf-2* RNAi; however, the knockdown of *gtr-1* did not mitigate proteotoxicity in day-4-old animals. (**B** and **C**) The knockdown of *gtr-1* enhances the number of polyQ35-YFP containing foci in day-6-old, but not in 4-day-old, AM140 worms (scale bar, 300 μm). (**D**) Separation of polyQ35-YFP structures using native agarose gels indicates that the knockdown of *gtr-1* enhances the amounts of high aggregated polyQ35-YFP at the expense of oligomers, at day-6-old animals, but much less prominently in day-4-old worms. (**E** and **F**) AM140 worms were grown from hatching on *gtr-1* RNAi and subjected to the filter-trap assay. The knockdown of *gtr-1* enhanced the aggregation of polyQ35-YFP in day-6-old animals but not in the day-4-old animals (E). This effect is reproducible and significant (F). (**G** and **H**) WB analysis using an Aβ antibody (clone 6E10) shows that the knockdown of *gtr-1* greatly reduces Aβ aggregation (G). Three independent assays as in (G) confirm the significance of this effect (H) [**P < 0.01, ***P < 0.001, ****P < 0.0001. Significance in (A), (C), and (F) was calculated using two-way analysis of variance (ANOVA) test, in (H) using one-way ANOVA test.]. n.s., not significant.

To further test the prediction that the mitigation of polyQ35-YFP–mediated proteotoxicity emanates from the knockdown of *gtr-1* in neurons, we crossed AM140 animals with worms that process RNAi primarily in neurons (strain TU3401). The resulting animals were grown from hatching on *gtr-1* RNAi or left untreated and subjected to a thrashing assay on day 6 of adulthood. Our results (fig. S1C) show that the knockdown of *gtr-1* in these animals mitigate that toxicity of polyQ35-YFP in muscle cells. Similarly, substantial protection from proteotoxicity was obtained when day-6-old AM141 worms, which express polyQ40-YFP stretches in their muscles (*12*), were treated with *gtr-1* RNAi and subjected to the thrashing assay (fig. S1D).

To further scrutinize the possible role of *gtr-1* in mitigating polyQ35-YFP–mediated proteotoxicity and to assess the possibility of RNAi off-target effect, we crossed AM140 animals with worms that harbor a deletion in the sequence of *gtr-1* (strain tm6094). This deletion and *gtr-1* RNAi treatment similarly reduce the survival rates of wild-type worms at an elevated temperature (35°C, fig. S1E), implying that the deletion in the sequence of *gtr-1* impairs GTR-1 functionality. The resulting animals that express polyQ35-YFP in their muscles and lack functional GTR-1 (strain EHC161), were subjected to thrashing assays at days 4 and 6 of adulthood. Our results indicate that the deletion in *gtr-1* alleviates the thrashing impairment of polyQ35-YFP–expressing worms at both ages (fig. S1F).

It is noteworthy that we tried to rescue the low proteotoxic sensitivity typical to EHC161 worms by injecting these worms with a plasmid that drives the expression of *gtr-1* under its natural promoter region [3000 base pairs (bp) upstream of the gene’s open reading frame]. However, the transgenic animals did not transmit this transgene to their progeny. This suggests that similarly to other GPCRs (*26*), the overexpression of *gtr-1* may impair reproduction.

These observations indicate that the counter-proteotoxic effect of *gtr-1* knockdown is not Aβ specific, as reducing the activity of *gtr-1* by RNAi or deletion mitigates the toxicity of polyQ35-YFP and polyQ40-YFP stretches. They also indicate that the knockdown of *gtr-1* in neurons alleviates the toxicity of Aβ and polyQ stretches in muscles.

Both disaggregation and hyperaggregation are protective activities that sequester highly toxic oligomers from the cellular environment (*11*). Thus, we sought to explore whether the knockdown of *gtr-1* mitigates the toxicity of polyQ35-YFP by promoting hyperaggregation or by mediating disaggregation. First, we tested how the knockdown of *gtr-1* affects the number of polyQ35-YFP–containing foci, structures that are correlated with polyQ35-YFP aggregation (*12*). AM140 animals were either left untreated (EV) or grown from hatching on *gtr-1* RNAi bacteria. Visualization by fluorescent microscopy and foci quantification at days 4 and 6 of adulthood indicated that the number of polyQ35-YFP–containing foci is much higher in day-6-old *gtr-1* RNAi–treated animals compared with their age-matched untreated counterparts (EV) (Fig. 1, B and C). Nonetheless, no such increase was observed in day-4-old *gtr-1* RNAi–treated worms. These results suggest that the knockdown of *gtr-1* induces hyper polyQ35-YFP aggregation in midlife. To further scrutinize this theme, we used two complementary techniques. First, AM140 worms were cultured on either control (EV) or *gtr-1* RNAi bacteria and harvested at either days 4 or 6 of adulthood, and the rates of polyQ35-YFP aggregation were compared using native agarose gel electrophoresis (NAGE) (*27*). While reduced levels of polyQ35-YFP monomers were observed in day-4-old worms, no substantial difference in the amounts of oligomers was seen. In contrast, reduced amounts of oligomers and apparent elevated levels of highly aggregated polyQ35-YFP were detected in day-6-old worms that were treated with *gtr-1* RNAi (Fig. 1D and fig. S1G). In addition, we used the filter-trap assay (*28*) and found that the knockdown of *gtr-1* by RNAi enhances the rate of polyQ35-YFP aggregation at day-6-old worms but not in younger animals (Fig. 1, E and F). These results further support the notion that the knockdown of *gtr-1* protects 6-day-old AM140 worms from proteotoxicity by enhancing polyQ35-YFP aggregation in midlife.

We next asked whether hyperaggregation is also the mechanism by which *gtr-1* RNAi protects CL2006 worms that express Aβ in their muscles (*29*) from the toxicity of this aggregative peptide. CL2006 animals were grown on either *gtr-1* RNAi bacteria or left untreated (EV) and harvested at day 3 of adulthood, and levels of Aβ aggregation were compared by Western blot (WB) analysis. Contradictory to the effect of *gtr-1* RNAi on polyQ35-YFP aggregation, this treatment has led to reduced Aβ aggregation rates (Fig. 1, G and H). These observations suggest that distinct proteostasis promoting activities are acted upon the knockdown of *gtr-1*, to mitigate proteotoxicity that emanates from the aggregation of polyQ35-YFP and of Aβ. They also raise the question of whether the same transcription factors are involved in the alleviation of polyQ35-YFP– and Aβ-mediated toxicity upon the knockdown of *gtr-1.*

### Aging-regulating transcription factors are crucial for the counter-proteotoxic effect of *gtr-1* RNAi

The aforementioned, IIS-regulated, aging-controlling transcription factors DAF-16, SKN-1, and HSF-1 regulate the rate of Aβ aggregation in CL2006 worms (*11*, *17*). Thus, we wondered whether these transcription factors, as well as PQM-1, are needed for *gtr-1* RNAi–mediated counter-proteotoxic effect. Since the dilution of *gtr-1* RNAi lowers its effectiveness as an alleviator of Aβ proteotoxicity (fig. S2A), we created dual silencing cassettes to concurrently knock down the expression of *gtr-1* and of one of the four transcription factors (fig. S2B). CL2006 worms were fed with bacteria that express one of the dual silencing cassettes and subjected to a paralysis assay. Three independent paralysis assays indicate that a concomitant knockdown of *gtr-1* and *skn-1* prevents the knockdown of *gtr-1* from mitigating the toxicity of Aβ (Fig. 2A). In contrast, animals that were treated with the RNAi cassette that simultaneously knocks down *gtr-1* and *daf-16* exhibited lower rate of paralysis compared with their counterparts that were solely treated with *daf-16* RNAi (Fig. 2B), indicating that DAF-16 is dispensable for the mitigation of Aβ proteotoxicity by *gtr-1* RNAi. Similarly, a concurrent knockdown of *hsf-1* and *gtr-1* indicated that HSF-1 is not needed for *gtr-1* RNAi to reduce Aβ toxicity (Fig. 2C). As expected, when we separately knocked down the expression of *gtr-1* or *pqm-1*, an analogous reduction in the rate of Aβ-mediated paralysis was observed. However, unexpectedly, a simultaneous knockdown of *gtr-1* and *pqm-1* enhanced the paralysis phenotype (Fig. 2D). This puzzling observation suggests that these two genes negatively interact.

**Fig. 2.**
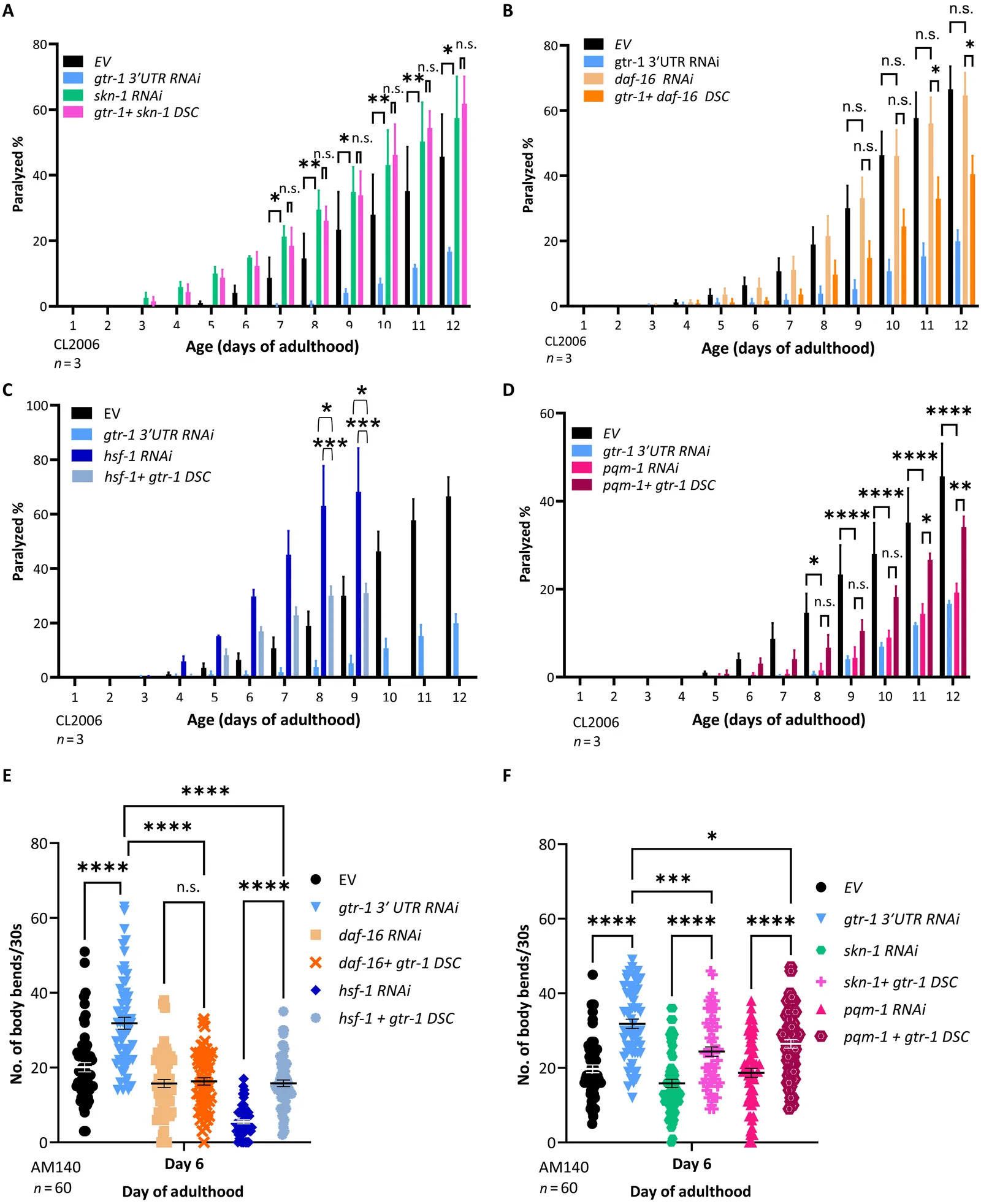
SKN-1 is needed for the mitigation of Aβ-mediated toxicity, and DAF-16 reduces the toxicity of polyQ35-YFP. (**A** to **C**) Three independent paralysis assays using CL2006 worms show that concurrent knockdown of *gtr-1* and of *skn-1* averts *gtr-1* RNAi from alleviating the toxicity of Aβ (A). In contrast, the knockdown of *gtr-1* mitigated proteotoxicity of Aβ when simultaneously knocked down with *daf-16* (B) or *hsf-1* (C). (**D**) RNAi toward *pqm-1* or *gtr-1* similarly mitigates Aβ-mediated proteotoxicity. However, a simultaneous knockdown of both genes enhanced the rate paralysis, indicative of elevated toxicity of Aβ. (**E** and **F**) Concomitant knockdown of *daf-16* and *gtr-1* prevented the knockdown of *gtr-1* from reducing the toxicity of polyQ35-YFP, as measured by the thrashing assay (E). On the contrary, the knockdown of *hsf-1* (E), *skn-1* or *pqm-1* (F) did not avoid *gtr-1* RNAi–mediated alleviation of polyQ35-YFP toxicity (DSC, dual-silencing cassette) [*P < 0.05, **P < 0.01, ***P < 0.001, ****P < 0.0001. Significance in (A) to (D) was calculated using two-way ANOVA test, in (E) and (F) using one-way ANOVA test].

Next, we wondered which transcription factors are needed for *gtr-1* RNAi to counter the toxicity of polyQ35-YFP. AM140 worms were treated from hatching with RNAi toward either *gtr-1*, *daf-16*, *skn-1*, *hsf-1*, or *pqm-1* or with one of the RNAi cassettes that concurrently target *gtr-1* and one of the four transcription factors. At day 6 of adulthood worms of all groups were subjected to thrashing assays, side by side with age-matched untreated animals. Our results (Fig. 2, E and F) indicate that the knockdown of *daf-16*, but none of the tested transcription factors obliterates the counter–polyQ35-YFP proteotoxicity effect of *gtr-1* RNAi.

To further test the roles of *daf-16* and of *skn-1* in the counter-proteotoxic effect that emanates from the knockdown of *gtr-1*, we used EHC161 worms (expressing polyQ35-YFP in their muscles and carry a deletion in *gtr-1*). The worms were either grown on control bacteria (EV) or treated with *daf-16* or *skn-1* RNAi. At day 6 of adulthood, all worm groups were subjected to thrashing assays side by side with AM140 animals. As expected, EHC161 worms exhibit higher thrashing rate than their age-matched AM140 counterparts. This protection from polyQ35-YFP was abolished by the knockdown of *daf-16* but not of *skn-1* (fig. S2C), confirming that *daf-16* is needed for the mitigation of polyQ proteotoxicity by the knockdown of *gtr-1.*

Collectively, our results indicate that two different transcription factors are needed for *gtr-1* RNAi–mediated protection from proteotoxicity. While SKN-1 activity is needed to mitigate the toxicity of Aβ, DAF-16 is involved in alleviating polyQ35-YFP–mediated proteotoxicity by *gtr-1* RNAi. This finding proposes that the expression levels of distinct gene sets are modulated by *gtr-1* RNAi according to the proteotoxic challenge that is faced by the worm muscles.

### Differential gene expression modulations in *gtr-1* RNAi–treated aβ- and polyQ35-YFP–expressing worms

Given that different transcription factors are needed for the counter-proteotoxic effect of *gtr-1* RNAi, we next sought to compare the *gtr-1* RNAi–induced transcriptomic modulations in worms that express Aβ and those that express polyQ35-YFP. First, we tested how the knockdown of *gtr-1* affects the transcriptomic landscape of Aβ-expressing worms. CL2006 were treated for two generations with *gtr-1* RNAi. Animals of the second generation were harvested at day 6 of adulthood, and the expression profiles of protein-coding genes were compared using RNA sequencing [RNA-seq; data are available at Gene Expression Omnibus (GEO): GSE296339]. The knockdown of *gtr-1* has led to modulations of gene expression compared to control, untreated animals (Fig. 3A), as 107 exhibited significant decrease (log2 < −0.58, *P* < 0.1), and 281 genes showed elevated expression levels in *gtr-1* RNAi–treated animals (log2 > 0.58, *P* < 0.1; Fig. 3A and fig. S3A).

**Fig. 3.**
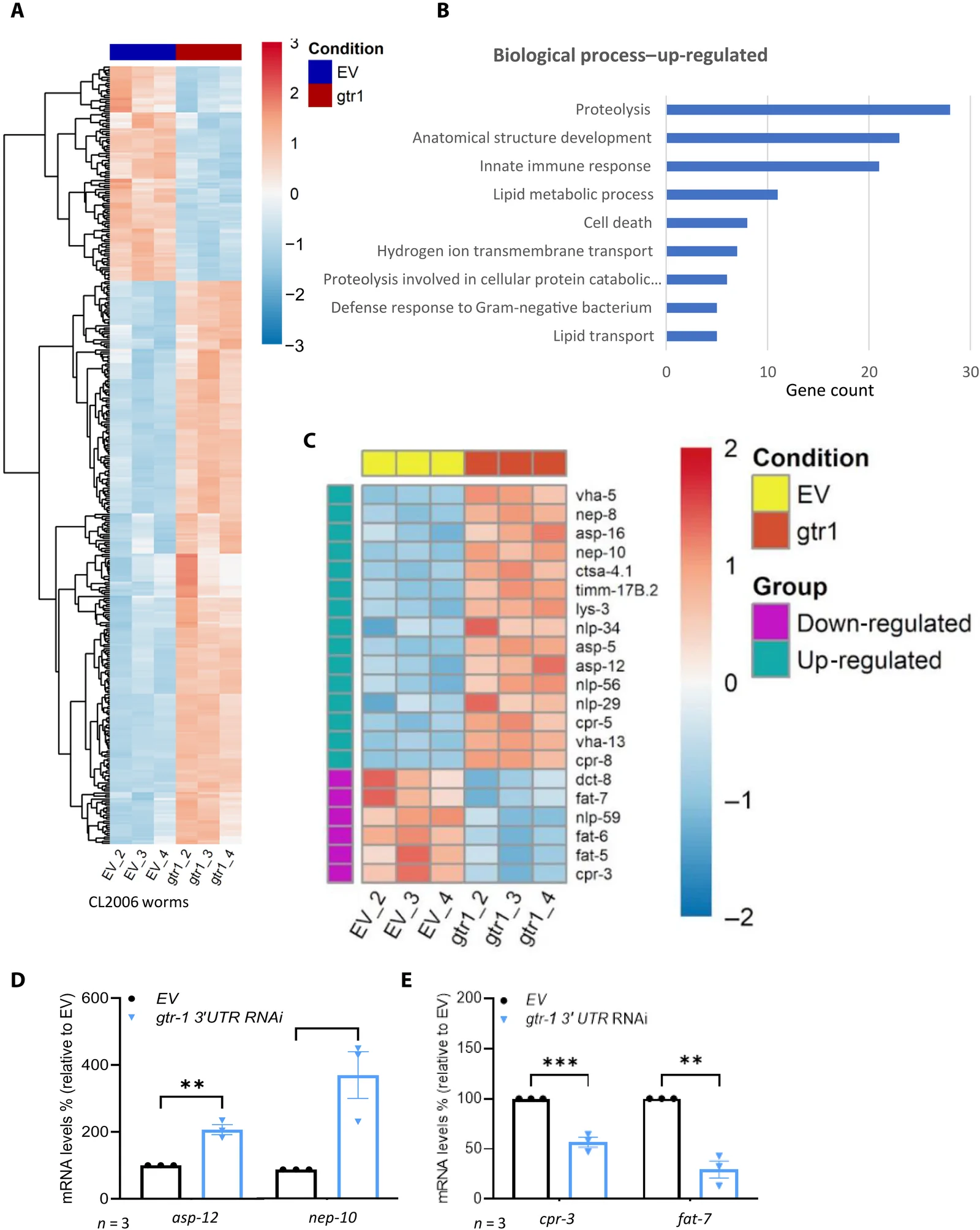
*gtr-1* RNAi treatment modifies the expression of proteolysis-associated and neuropeptide-coding genes in CL2006 worms. (**A**) A heatmap of RNA-seq results indicates that the knockdown of *gtr-1* modifies gene expression, whereas 107 genes exhibited reduced and 281 elevated expression levels compared with untreated day-6-old CL2006 animals. (**B**) Using the DAVID bioinformatic clustering tool, we clustered the affected genes and found that proteolysis-related genes are most prominent among the genes that show modulated expression levels. (**C**) A heatmap of a selected set of affected genes shows that proteases- and neuropeptide-coding genes are affected by the knockdown of *gtr-1.* (**D** and **E**) qPCR confirm that *asp-12* and *nep-10* exhibit elevated expression (D) and *cpr-1* and *fat-7* show reduced expression (E), upon the knockdown of *gtr-1* in CL2006 worms (*P < 0.05, **P < 0.01, ***P < 0.001. Significance in (D) and (E) was calculated using *t* tests.).

Clustering the differentially expressed genes (DEGs) according to biological processes, we found that genes that encode proteins that are involved in proteolysis are most prominent among the up-regulated genes (Fig. 3B). Kyoto Encyclopedia of Genes and Genomes (KEGG) analysis pointed at lysosomal genes as the second most prominent group of affected genes (fig. S3B). Similar results were obtained when we used WormCat to cluster the affected genes, as genes which are involved in stress response and in proteolysis were most prominently affected by the knockdown of *gtr-1* (fig. S3C).

A detailed look at the genes that have shown modulated expression levels (Fig. 3C) highlights two proteostasis-related gene groups. A group of genes that are involved in proteolysis including *nep-8* and *nep-10*, both encoding metallopeptidases of the neprilysin family, are up-regulated in *gtr-1* RNAi–treated CL2006 animals. This is an interesting finding as an overexpression of neprilysin has been shown to protect AD-model mice from neurodegeneration (*30*). The aspartyl proteases coding genes *asp-12* and *asp-16*, as well as members of the cathepsin family of proteases *cpr-5* and *cpr-8*, also show elevated expression levels, suggesting that lysosomal proteolysis is enhanced in worms that express low *gtr-1* levels. *cpr-3*, a gene that encodes a protease member of the cathepsin B family, exhibits low expression levels when *gtr-1* was knocked down. This is not particularly unexpected as cathepsin B was shown to enable the onset of neurodegeneration in AD mouse models, and the knockout of this gene alleviates behavioral deficits in these animals (*31*). Likewise, we recently found that the knockdown of *cpr-6* that encodes a cathepsin B protects CL2006 from proteotoxicity, as measured by the paralysis assay (*32*). Quantitative real-time polymerase chain reaction (qPCR) using specific primers toward the protease-coding genes, *nep-10* and *asp-12*, confirmed the RNA-seq results, as both genes exhibited significantly increased expression levels in *gtr-1* RNAi–treated worms compared with control, untreated animals (Fig. 3D). Similarly, the expression levels of *fat-7* and *cpr-3* were significantly reduced upon the knockdown of *gtr-1* as predicted by our RNA-seq experiment (Fig. 3E).

An additional interesting group of genes that displayed modulated expression levels upon the knockdown of *gtr-1* are genes that encode for neuropeptides. These include *nlp-29*, *nlp-34*, which are foremost expressed in the hypodermis (*33*), and *nlp-56*; all show elevated expression levels in *gtr-1* RNAi–treated worms. In contrast, *nlp-59* exhibited reduced expression level upon the knockdown of *gtr-1* (Fig. 3C). This finding is of particular interest as neuropeptide signaling has been reported to regulate endoplasmic reticulum stress resistance and longevity (*34*) and to differentially control the organismal response to distinct proteotoxic challenges (*24*). It also raises the prospect that these molecules are among the messengers that carry the signal from the neuronal system to distal cells to orchestrate proteostasis.

Next, we asked how the knockdown of *gtr-1* affects the transcriptomic landscapes of animals that express polyQ35-YFP in their muscles. AM140 animals were either left untreated or fed for two generations with *gtr-1* RNAi bacteria, harvested at day 6 of adulthood, and subjected to RNA-seq (data are available at GEO: GSE318419). Four hundred seventy-one genes exhibited increased expression levels (log2 > 0.58, *P* < 0.1) upon the knockdown of *gtr-1*, and 742 showed reduced expression (log2 < −0.58, *P* < 0.1; Fig. 4A and fig. S4A). Among the genes that exhibited elevated expression levels in *gtr-1–*treated AM140 animals, we identified genes that also showed increased expression in CL2006 animals. These include the protease-coding genes *nep-8*, *nep-10*, *cpr-5*, and *cpr-8* (Fig. 4B). The increased expression of *nep-*10 in *gtr-1* RNAi–treated AM140 worms was validated by qPCR (Fig. 4C). These observations indicate that certain proteostasis-associated genes are similarly affected by *gtr-1* RNAi in both proteotoxicity model strains and suggest that they are generally needed to mitigate proteotoxicity of distinct aggregation-prone proteins. Proteases appear to be abundant in this group. In contrast, the expression of *cav-1*, which is crucial for the formation of caveolae, and was shown to impair proteostasis (*35*), was reduced in *gtr-1* RNAi–treated AM140 animals but not in their Aβ-expressing counterparts. An additional interesting gene that exhibits reduced expression in *gtr-1* RNAi–treated worms is *cyn-17*, which encodes a chaperone, a member of the cyclophilin family. The malfunction of cyclophilins was reported to underlie the misfolding of neurodegeneration-causing proteins (*36*, *37*).

**Fig. 4.**
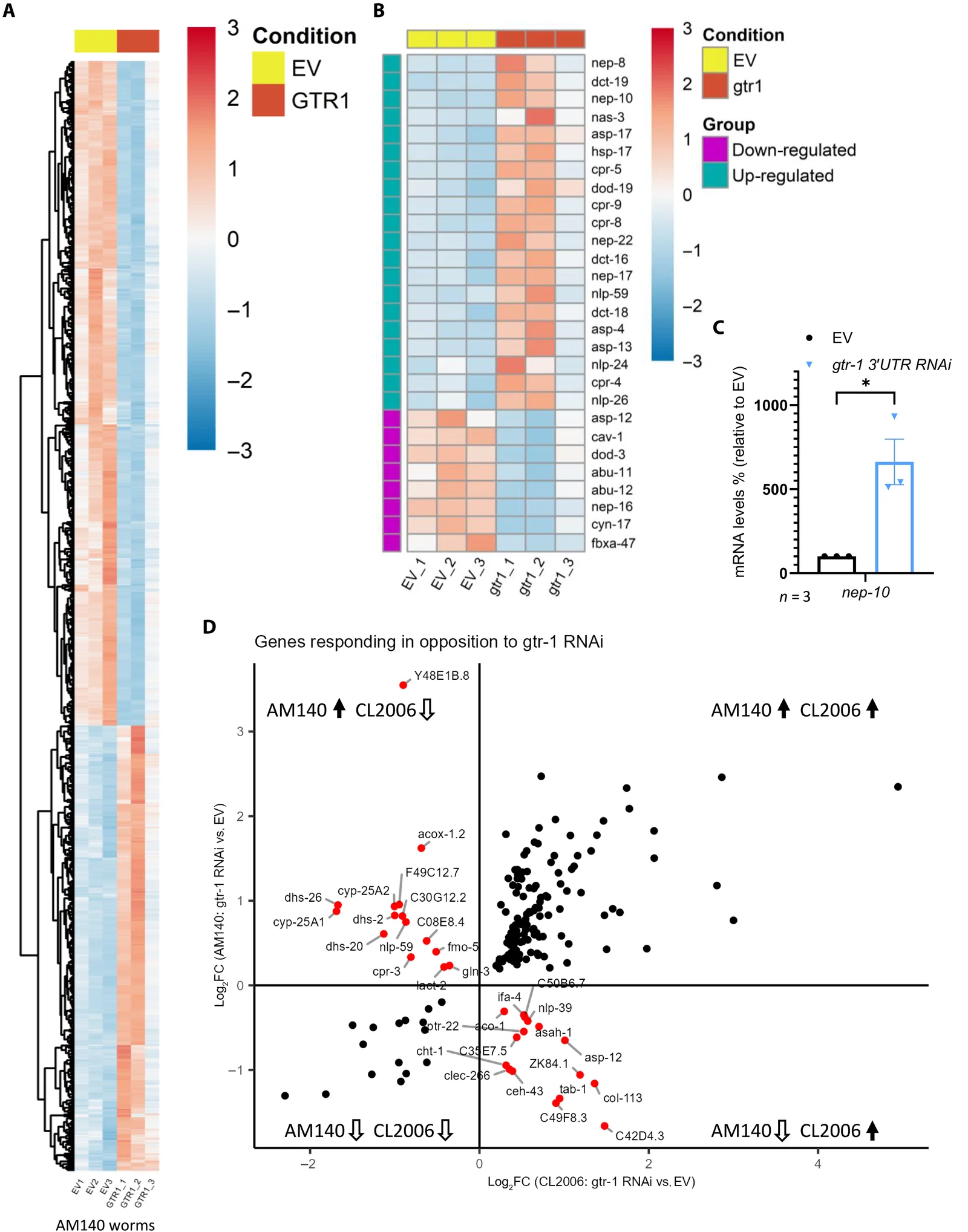
The knockdown of *gtr-1* modulates the transcriptomic landscape of AM140 worms. (**A**) A heatmap shows significant differences in genes expression in untreated (EV) and *gtr-1* RNAi–treated, day-6-old AM140 worms, as 471 genes exhibited elevated and 742 showed reduced expression levels. (**B**) A heatmap of a selected subset of genes shows that genes that encode certain proteases, neuropeptides, and known proteostasis-regulating proteins show changes in expression levels upon the knockdown of *gtr-1.* (**C**) qPCR validated that *nep-10* displays significantly elevated expression in *gtr-1* RNAi–treated AM140 worms. (**D**) A comparison of the RNA-seq experiments unveils a subset of genes that respond in an opposing manner to the knockdown of *gtr-1* in CL2006 and AM140 worms. These genes include the neuropeptide-coding genes *nlp-39* and *nlp-59*, as well as the protease-coding genes *asp-12* and *cpr-3* (*P < 0.05. Significance in (C) was calculated using *t* tests.).

Clustering the genes that exhibit modulated expression levels in AM140 worms according to their involvement in biological processes, we found that genes that have roles in lipid metabolism and lipid biosynthesis are most prominent among the up-regulated genes (fig. S4B), and those that are involved in transcription are most abundant among the down-regulated (fig. S4C). These results were very different than those that we obtained using CL2006 animals where proteolysis-promoting genes were more prominent (fig. S3B).

Since DAF-16 was found to be critically needed for the mitigation of polyQ35-YFP proteotoxicity (Fig. 2E), we wondered whether genes that are known to be regulated by this transcription factor are enriched among the genes that show modulated expression levels in *gtr-1* RNAi–treated AM140 worms. To test this, we clustered the genes that exhibited significantly modulated expression levels in our RNA-seq experiment, according to the transcription factors that are known to regulate their expression levels. In both up-regulated and down-regulated genes, the most prominent groups were of DAF-16–controlled genes (fig. S4, D and E, respectively).

To further elucidate the relations between DAF-16 and GTR-1, we compared the lists of DAF-16–regulated genes (*38*) that show modulated expression levels in *gtr-1* RNAi–treated CL2006 and AM140 animals. First, we focused on DAF-16 target genes that respond concordantly in the two worm strains (fig. S4F) and found among them the protease-coding gene *nep-8* as well as *dct-19*, which is predicted to be involved in the innate immune response. Next, we used temperature-sensitive feminized worms (strain CF512) to compare the expression levels of specific DAF-16 target genes by qPCR. When exposed to 25°C during development, these worms produce no sperm and have no progeny, enabling the comparison of gene expression in somatic adult tissues, avoiding potential background from developing embryos. CF512 worms were treated from hatching with RNAi toward either *gtr-1* or *daf-16*, and the levels of two known DAF-16 target genes, *mtl-1* and *sod-3* (*39*), were compared by qPCR to these of untreated control animals (EV). Our results (fig. S4, G and H) show that these two genes responded concordantly to the RNAi-mediated knockdown of *gtr-1* and *daf-16.* We next plotted DAF-16 targets that respond in opposition to the knockdown of *gtr-1* in CL2006 and AM140 worms (fig. S4I). An interesting gene in this group is the neuropeptide-coding gene *nlp-39*. These results further show that the knockdown of *gtr-1* differentially affects gene expression profiles in worms that are challenged by the expression of distinct proteotoxic proteins and further highlight the roles of neuropeptides in this challenge-specific proteostasis network’s response. Last, we examined whether the knockdown of *gtr-1* reduces the expression of *daf-16.* To address this, we used worms that express DAF-16 fused to green fluorescent protein (GFP) (strain CF1934). The worms were left untreated (EV) or treated with *gtr-1* RNAi and visualized at day 1 of adulthood. Our results clearly show that *gtr-1* RNAi greatly reduces the levels of DAF-16::GFP (fig. S4J).

The second most prominent transcription factor in the clustering group of up-regulated genes is PQM-1 (fig. S4D), whose knockdown does not prevent *gtr-1* RNAi from mitigating polyQ35-YFP proteotoxicity (Fig. 2F). This observation suggests that PQM-1 may be involved in other functions of GTR-1.

To further test how the expression of distinct proteotoxic proteins differentially affects the worm’s transcriptome, we searched for genes that respond in opposition to the knockdown of *gtr-1* in AM140 and CL2006 animals (Fig. 4D). Among the opposingly responding genes, we identified two protease coding genes, *asp-12* and *cpr-3.* This finding supports the notion that certain aspects of the proteolysis modulation by *gtr-1* RNAi are proteotoxic challenge specific. Two neuropeptide-coding genes, *nlp-59* and *nlp-39*, also exhibited opposing modulations. While *nlp-59* exhibits elevated expression in AM140 animals, it shows reduced expression in CL2006 worms. *nlp-39* shows an opposing expression pattern in the two strains, reduced level in AM140 worms and elevated expression in CL2006 animals. These findings suggest that, as shown previously (*24*), certain neuropeptides opposingly affect proteostasis; while aggravating the toxicity of one proteotoxic protein, they alleviate the toxicity of another aggregation-prone protein. Thus, we set out to directly test how *gtr-1* RNAi affects proteolysis and examine how neuropeptides influence proteotoxicity that emanates from the two proteotoxic proteins, Aβ and polyQ35-YFP.

### Reducing the expression of *gtr-1* enhances proteolysis

To scrutinize the prediction that the knockdown of *gtr-1* enhances proteolysis, we first compared the rates of UPS activity in CF512 worms. These animals were used to avoid possible background of UPS activity that may stem from developing embryos. The worms were either left untreated (EV) or treated with *gtr-1* RNAi and homogenized at day 3 of adulthood, and each homogenate was split into two samples. One was supplemented with the UPS inhibitor MG132, while the other was left with no supplements. The rates of UPS activity were measured using the fluorogenic proteasome substrate Suc-LLVY-AMC, which enables the measurement of chymotrypsin-like proteasome activity (Fig. 5A). Three independent experiments indicated that the knockdown of *gtr-1* enhances the rate of chymotrypsin-like proteasome activity (Fig. 5B).

**Fig. 5.**
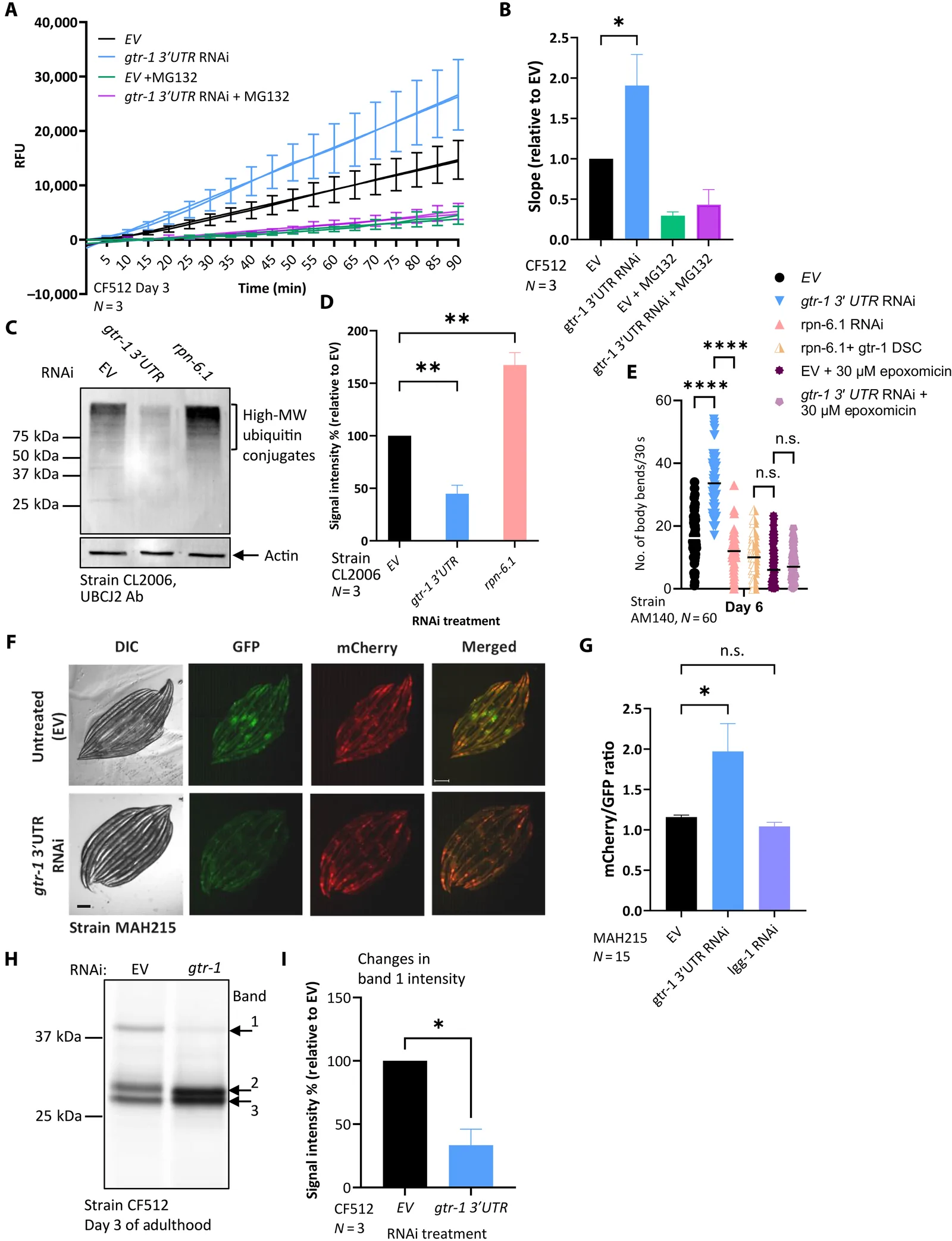
Protein degradation mechanisms are activated by *gtr-1* RNAi. (**A** and **B**) An in vitro, Suc-LLVY-AMC–based assay indicates the knockdown of *gtr-1* by RNAi enhances chymotrypsin-like proteasome activity in CF512 animals. The proteasome activity inhibitor MG132 was used as a negative control (A). Three independent repeats show that this effect is significant (B). (**C** and **D**) CL2006 worms were treated from hatching with RNAi toward *gtr-1* or *rpn-6.1* (the latter used to inhibit UPS activity), harvested at day 3 of adulthood, and subjected to WB using an antibody that interacts with ubiquitinated proteins (clone UBCJ2). Our results (C) show that the knockdown of *gtr-1* reduces the amount of high-MW Ub, indicative of enhanced UPS activity. Three independent repeats confirmed the significance of UPS activity enhancement upon the knockdown of *gtr-1.* (**E**) A thrashing assay using day-6-old AM140 animals whose UPS activity was inhibited by treatment either with *rpn-6.1* RNAi or with the proteasome inhibitor epoxomicin show reduced thrashing rates regardless of whether they were treated with *gtr-1* RNAi or not. (**F** and **G**) The rates of autolysosome formations in untreated (EV) and *gtr-1* RNAi–treated worms were compared using animals that express the chimeric protein mCherry::GFP::lgg-1, whereas the GFP is sensitive to acidic conditions. The knockdown of *gtr-1* has led to an increased ratio of mCherry/GFP indicative of elevated autolysosome formation [(F) scale bar, 150 μm]. Fluorescence quantification in 15 worms per treatment confirmed the significance of this phenotype (G). (**H** and **I**) A comparison of CPR-6 activity using the probe GB123 shows that the knockdown of *gtr-1* modifies the signature of this activity in day-3-old CF512 worms (H) in a reproducible and significant manner (I) [*P < 0.05, **P < 0.01, ****P < 0.0001. Significance in (A) and (B) was calculated using two-way ANOVA test. In (D), (E), and (G), significance was calculated using one-way ANOVA and in (I) using *t* test].

To further test whether the knockdown of *gtr-1* enhances UPS activity, we compared the amounts of high–molecular weight ubiquitin conjugates (high-MW Ub) in untreated CL2006 worms and in their counterparts that were treated with RNAi toward either *gtr-1* or *rpn-6.1* [the latter RNAi is known to inhibit proteasome activity (*40*)]. The worms were harvested at day 3 of adulthood, and rates of ubiquitin conjugates were compared by WB using an antibody that reacts with ubiquitin-conjugated proteins. Homogenates of worms that were treated with *gtr-1* RNAi contained much less high-MW Ub conjugates compared with homogenates of untreated and of *rpn-6.1* RNAi–treated animals (Fig. 5, C and D), confirming that the knockdown of *gtr-1* enhances UPS activity.

Similar results were obtained when we used a third technique that is based on animals that were engineered to express a short-lived chimeric protein that consists of ubiquitin fused to the GFP (Ub^G76V^-GFP, strain PP563). When the UPS functions properly, this chimeric protein is rapidly degraded after synthesis; nevertheless, a certain level of fluorescence can be detected, foremost in the intestine of these nematodes. Enhanced UPS activity reduces the fluorescent signal in these worms (*41*). As a control, we used PP545 worms that express a Ub^G76V^-GFP that carries two mutations in the ubiquitin sequence, K29R and K48R. These mutations avert the binding of additional ubiquitin peptides, thereby preventing the direction of the chimeric protein for proteasomal degradation. PP563 and PP545 worms were cultured on bacteria that express RNAi toward *gtr-1* or left untreated (EV). At day 1 of adulthood, the animals were visualized by fluorescent microscopy (fig. S5A) and subjected to WB analysis using a GFP antibody (fig. S5, B and C). Both microscopic visualization and WB analysis indicated that the knockdown of *gtr-1* by RNAi reduces the GFP fluorescence level compared with the level seen in untreated PP563 animals (EV) but not in PP545 worms. These results further validate our conclusion that the knockdown of *gtr-1* elevates UPS activity and raise the question of whether enhancing this activity is vital for the counter-proteotoxic effect of *gtr-1* RNAi. To address this, we concurrently knocked down the expression of *gtr-1* and inhibited UPS activity using a dual-silencing RNAi cassette toward *gtr-1* and *rpn-6.1* (*40*). Subjecting AM140 worms that were treated from hatching with the *gtr-1/rpn-6.1* cassette, to a thrashing assay, we found that the inhibition of UPS prevented *gtr-1* RNAi from mitigating polyQ35-YFP proteotoxicity (Fig. 5E). Similar results were obtained when day-6-old AM140 animals were treated with 30 μM of the irreversible UPS inhibitor epoxomicin (Fig. 5E). This epoxomicin concentration is sufficient to promote proteotoxicity in AM140 animals (fig. S5D). Together, these results confirm the assumption that UPS enhancement is vital for the *gtr-1* RNAi–mediated protection from proteotoxicity.

Since lysosomal proteases exhibit modified expression levels upon the knockdown of *gtr-1* (Figs. 3C and 4B), and autophagy being an additional key protein degradation mechanism that plays crucial roles in proteostasis maintenance, as well as in the prevention of neurodegeneration (*42*, *43*), we sought to test whether the knockdown of *gtr-1* also enhances the activity of this mechanism. To address this, we used worms that express a chimeric fluorescent protein that is composed of LGG-1 fused to mCherry and pH-sensitive GFP. When this protein is present in an acidic environment, GFP signal is quenched, but the signal of mCherry is not affected. Accordingly, when autophagy is enhanced, the ratio of mCherry signal to GFP fluorescence is elevated [strain MAH215 (*44*)]. MAH215 animals were either treated with *gtr-1* RNAi or left untreated and visualized by a dual-channel fluorescent microscope at day 1 of adulthood. Reduced GFP signal was seen in animals that were treated with *gtr-1* RNAi (Fig. 5, F and G), indicative of enhanced formation of autolysosomes and elevated levels of autophagy in these worms.

The modulated expression levels of genes that encode proteases of the cathepsin family upon the knockdown of *gtr-1* (Figs. 3C and 4B) have prompted us to test whether *gtr-1* RNAi affects the activity of the cathepsin B ortholog CPR-6 using the activity-based probe GB123 (*45*) that enables the measurement of CPR-6 activity in homogenates of *C. elegans* (*32*). Our results show that day-3-old CF512 animals that were treated from hatching with *gtr-1* RNAi exhibit a modulated CPR-6 activity pattern as the upper band (Fig. 5H, band 1) that is much weaker compared with the one seen in control animals. In contrast, the two lower bands (bands 2 and 3) are intensified. This notable change in CPR-6 activity pattern (Fig. 5I), is typical to young animals and associated with reduced proteotoxicity (*32*). Together, our results culminate to show that the knockdown of *gtr-1* greatly enhances protein degradation by elevating UPS activity, modulating the activity of cathepsin B and enhancing the formation of autolysosomes.

### Neuropeptides are needed for the promotion of proteostasis by *gtr-1* RNAi

How neurons communicate with somatic cells to promote proteostasis is a key question in the field. Since certain neuropeptides are involved in the coordination of proteostasis (*24*, *34*), we tested whether *nlp-29*, *nlp-34*, and *nlp-56*, whose expression levels were elevated in CL2006 worms, and *nlp-59*, which showed reduced expression in *gtr-1* RNAi–treated CL2006 animals (Fig. 3C) and elevated expression in AM140 animals (Fig. 4B), are needed for the counter-proteotoxic effect of *gtr-1* knockdown. To avoid reduction in the knockdown efficiency of *gtr-1* due to dilution (fig. S2A), we created dual-silencing RNAi cassettes that mediate a concomitant knockdown of *gtr-1* and of one of the aforementioned neuropeptide-coding genes (fig. S6A). Populations of CL2006 were grown from hatching on EV bacteria or on RNAi toward either one of the neuropeptides coding genes or on one of the dual-silencing cassettes. Paralysis assays indicated that the knockdown of all three neuropeptides that exhibited elevated levels upon the knockdown of *gtr-1*, namely, *nlp-29*, *nlp-34*, and *nlp-56*, mitigated the toxicity of Aβ (Fig. 6, A to C). Nevertheless, a concurrent knockdown of *gtr-1* and of these neuropeptide-coding genes did not enable *gtr-1* RNAi to fully protect the worms from Aβ proteotoxicity. In contrast, the knockdown of *nlp-59*, the sole neuropeptide that showed reduced expression level upon the knockdown of *gtr-1* in these worms, partially protected the worms from the paralysis phenotype but did not avert *gtr-1* RNAi from fully protecting the animals (Fig. 6D).

**Fig. 6.**
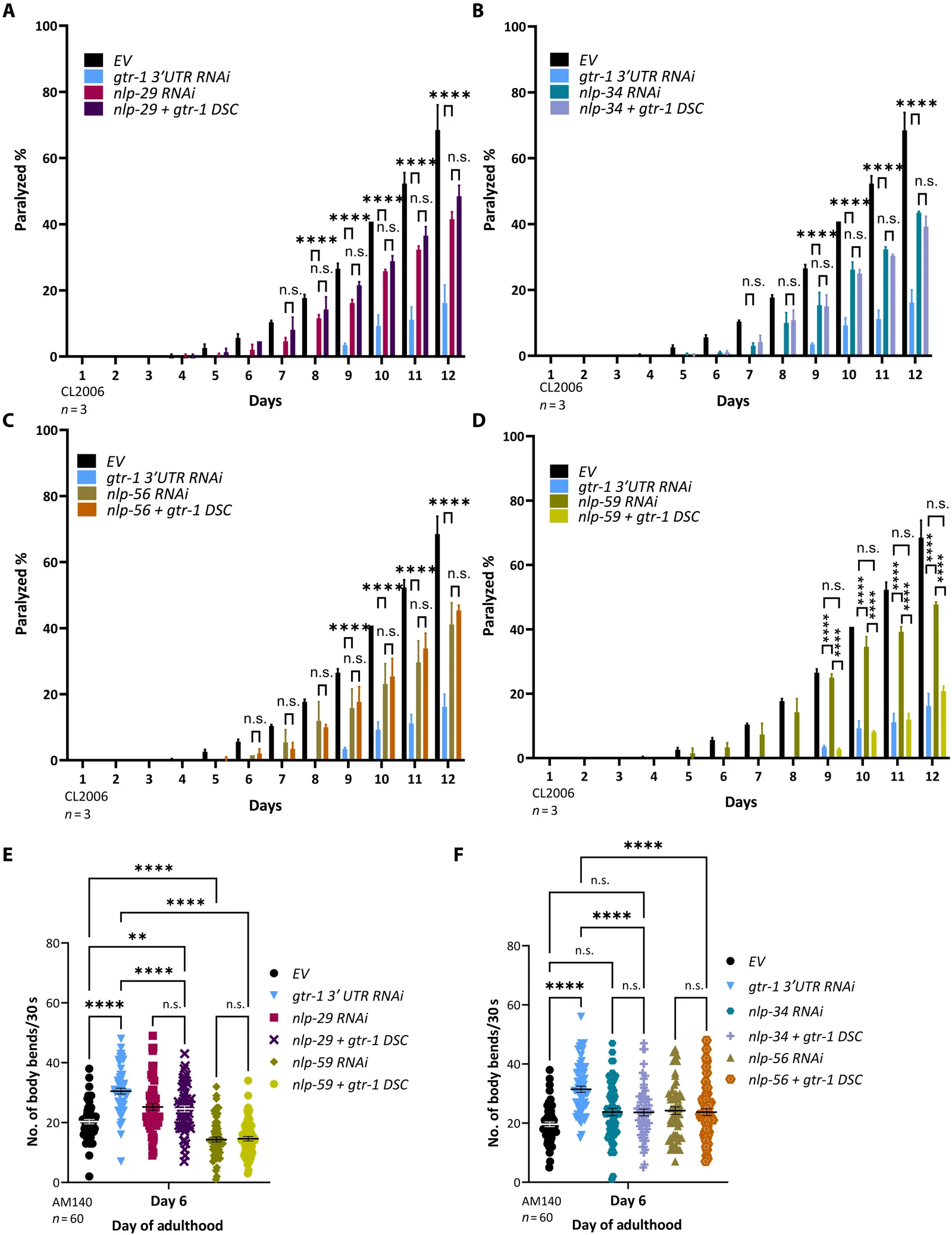
Neuropeptides are needed for the counter-proteotoxic effects of *gtr-1* RNAi. (**A** to **C**) Paralysis assays show that the knockdown of *nlp-29* (A), *nlp-34* (B), and *nlp-56* (C) mitigates the toxicity of Aβ but prevent the knockdown of *gtr-1* from providing its full counter-proteotoxic effect. The knockdown of *nlp-59* slightly reduces Aβ-mediated toxicity but allows *gtr-1* RNAi to execute its full protection from this aggregative peptide (**D**). (**E** and **F**) The knockdown of all tested neuropeptide-coding genes, *nlp-29*, *nlp-34*, *nlp-56*, and *nlp-59*, avert *gtr-1* RNAi from mitigating the toxicity of polyQ35-YFP. While RNAi toward *nlp-59* enhanced the toxicity of polyQ35-YFP, the knockdown of *nlp-29* slightly reduced this toxicity (E) [**P < 0.01, ****P < 0.0001. Significance in (A) to (D) was calculated using two-way ANOVA test and in (E) and (F) using one-way ANOVA.].

We next treated CL2006 worms from hatching with RNAi toward each of the tested neuropeptides or toward *gtr-1* or *Aβ*. At day 1 of adulthood, the worms were homogenized and subjected to WB analysis to compare Aβ levels. Our results confirm that as shown in Fig. 1G, the knockdown of *gtr-1* greatly reduces the levels of Aβ and show that the knockdown of any of the tested neuropeptides only moderately reduces the level of this aggregation-prone peptide (fig. S6B).

Using AM140 worms and the thrashing assay, we tested whether the knockdown of neuropeptides also affects the toxicity of polyQ35-YFP and found that while the knockdown of *nlp-29* mitigated proteotoxicity, its concomitant knockdown with *gtr-1* did not further protect the worms (Fig. 6E). In contrast to its effect on Aβ toxicity, the knockdown of *nlp-59* aggravated polyQ35-YFP–mediated toxicity, as a concurrent knockdown of *nlp-59* and of *gtr-1* did not rescue the worms from the low thrashing rate (Fig. 6E). The knockdown of *nlp-34* and of *nlp-56* had no effect on the rates of thrashing; nonetheless, it prevented *gtr-1* RNAi from alleviating the toxicity of polyQ35-YFP (Fig. 6F). It is important to note that the knockdown of either one of the aforementioned neuropeptide-coding genes by RNAi did not prominently affect the levels of polyQ35-YFP in day-1-old AM140 worms, as judged by fluorescent visualization (fig. S6C). These observations can be explained by two themes. First, these messengers may affect proteotoxicity in later stages of life, and thus, no change in the levels of polyQ35-YFP can be seen at day 1 of adulthood. Alternatively, the knockdown of one neuropeptide at a time is not sufficient to modulate the levels of this aggregative protein.

We next used CF1934 worms to test whether the knockdown of *gtr-1* or of the identified neuropeptides modulates the levels of DAF-16. The worms were either left untreated (EV) or fed from hatching with bacteria that express RNAi toward either *gtr-1* or one of the tested neuropeptide-coding genes. The worms were homogenized at day 3 of adulthood and subjected to WB analysis using a GFP antibody (fig. S6D). Three independent repeats indicated that the knockdown of *gtr-1*, but not of any of the neuropeptide-coding genes, reduces the levels of DAF-16::GFP by ∼50% (fig. S6E).

Last, we asked whether the knockdown of any of the tested neuropeptides is needed for the observed enhancement of polyQ35-YFP aggregation by *gtr-1* RNAi. AM140 worms were treated from hatching with RNAi toward one of the tested neuropeptides or with the dual-silencing cassettes that concurrently knock down *gtr-1* and one of the neuropeptide-coding genes and harvested at day 6 of adulthood, and the rates of SDS-resistant polyQ35-YFP aggregation were compared by the filter-trap assay. Our results (fig. S6, F and G) show that, while treatment with *gtr-1* RNAi enhances the aggregation of polyQ35-YFP (as seen in Fig. 1, E and F), the knockdown of any of the tested neuropeptides reduced this phenomenon regardless of whether the expression of the neuropeptide-coding gene was solely reduced or together with *gtr-1*.

## DISCUSSION

How neurons coordinate proteostasis across the organism is an enigmatic question that is only partially understood. We previously reported that knocking down the expression of *gtr-1*, which most likely encodes a neuronal GPCR, alleviates chronic proteotoxicity that is inflicted by Aβ expression in the worm’s body wall muscles. Here, we used the nematode *C. elegans* to investigate the proteostasis-promoting mechanism that is activated by *gtr-1* RNAi in two proteotoxicity model worm strains and found several key aspects of this neuron-to-soma proteostasis-promoting mechanism. We found that *gtr-1* RNAi treatment also alleviates polyQ35-YFP–mediated proteotoxicity when *gtr-1* is knocked down throughout the worm or primarily in neurons. It is important to note that while *gtr-*1 appears to be expressed in neurons (*23*) and that the knockdown of *gtr-1* in worms that were crossed with TU3401 animals shows protection from proteotoxicity, the question of whether *gtr-1* acts in neurons as a regulator of proteostasis requires further clarification. This stems from the expression of *gtr-1* in non-neuronal tissues such as pharyngeal muscles (*33*), Furthermore, a recent study unveiled that TU3401 exhibits residual RNAi processing in non-neuronal tissues (*46*). Thus, in the future, it will be important to rescue the expression of this gene solely in neurons of *gtr-1* mutant worms, to further test whether the neuronal activity of this receptor is sufficient to regulate proteotoxicity across the organism.

While the mitigation of polyQ35-YFP toxicity by *gtr-1* RNAi is associated with hyperaggregation of this protein, Aβ undergoes disaggregation upon the knockdown of *gtr-1.* Physiological RNAi-based experiments unveiled that the transcription factor SKN-1 is needed for protection from Aβ upon the knockdown of *gtr-1*, and DAF-16 is required for *gtr-1* RNAi–mediated alleviation of polyQ35-YFP proteotoxicity. RNA-seq experiments using Aβ- and polyQ35-YFP–expressing worms pointed at proteolysis-related genes as one of the most prominent gene groups that show modulated expression levels in CL2006 worms upon the knockdown of *gtr-1*. Activity assays confirmed that both proteasome activity and autolysosome formation rate are enhanced in *gtr-1* RNAi–treated CL2006 animals. Last, we found that four neuropeptides that exhibited modulated expression levels upon the knockdown of *gtr-1* are involved in the counter-proteotoxic effect of this treatment. Notably, while *nlp-29*, *nlp-34*, and *nlp-56* were found to be needed for the *gtr-1* RNAi–mediated protection from both Aβ and polyQ35-YFP, the knockdown of *nlp-59* was dispensable for the mitigation of Aβ toxicity but vital for the alleviation of polyQ35-YFP toxicity by *gtr-1* RNAi. Unexpectedly, despite the dispensability of *nlp-59* for the counter-proteotoxic effect of *gtr-1* RNAi (Fig. 6D), the knockdown of this neuropeptide appears to moderately reduce the total levels of Aβ (fig. S6B). However, it is unclear whether this limited modulation in the levels of Aβ has a functional meaning. Further research will be needed to clarify this ambiguity.

These results are consistent with the prediction of the RNA-seq experiments and further imply that distinct mechanisms are activated by *gtr-1* RNAi in the face of different proteotoxic challenges (Fig. 7). We already reported that neurons differentially respond to the expression of Aβ and polyQ35-YFP in muscles by secreting neuropeptides (*24*). However, is this the case when *gtr-1* is knocked down? The finding that *nlp-59* is required for *gtr-1* RNAi to protect AM140 worms (Fig. 6E), but not CL2006 animals (Fig. 6D), and the observation that this gene responds in opposition to the knockdown of *gtr-1* in AM140 and CL2006 worms (Fig. 4D) further support this notion. Studies by others have shown the involvement of neuropeptides in the orchestration of proteostasis across the organism (*34*), thereby supporting the possibility that GTR-1 governs neuropeptide signaling to differentially modulate proteotoxicity of distinct aggregation-prone proteins in remote tissues.

**Fig. 7.**
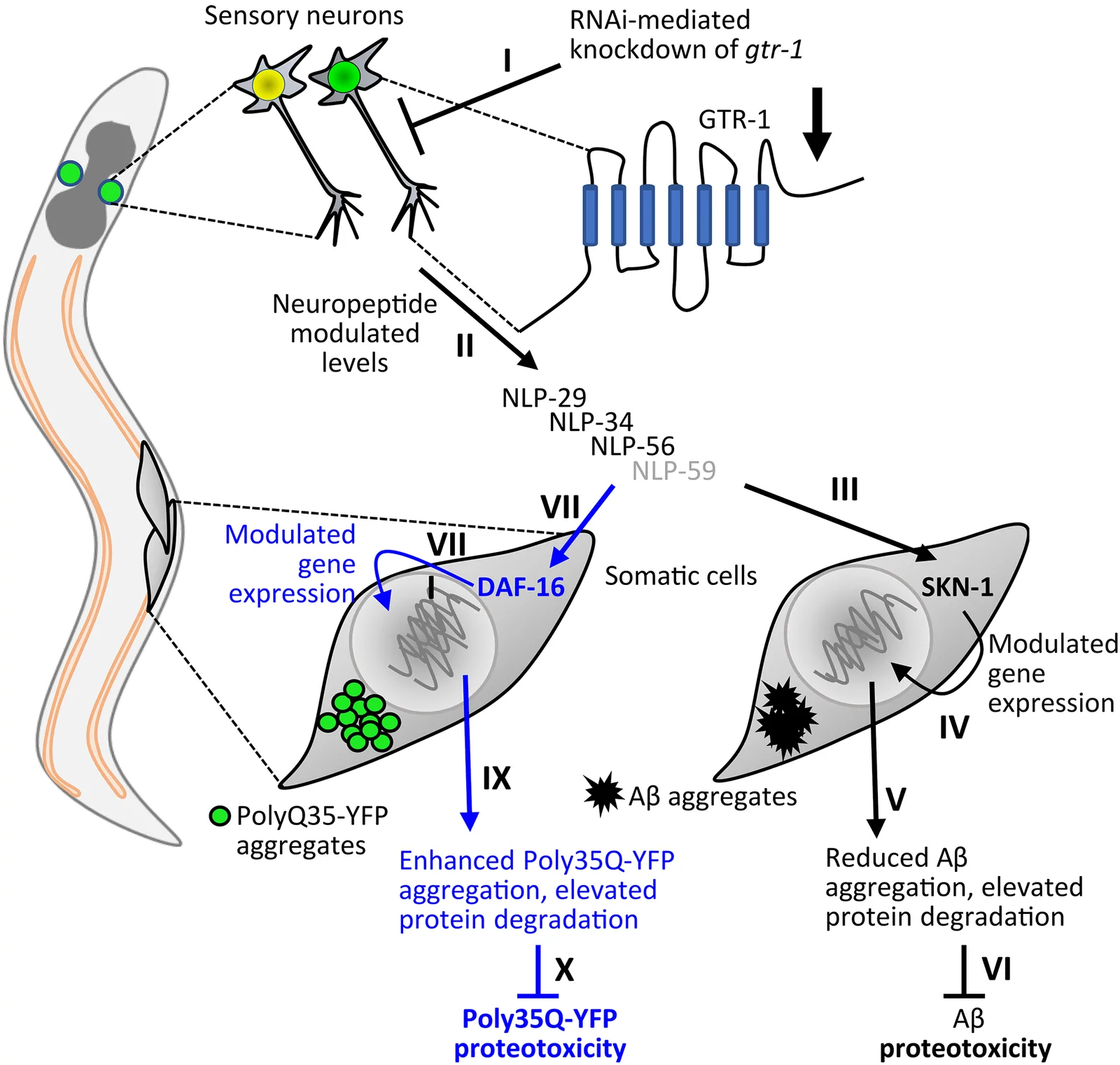
Distinct mechanisms are activated by *gtr-1* RNAi to counter Aβ and polyQ35-YFP–mediated proteotoxicity. The proposed model it based on the likely, but not proven, assumption that GTR-1 functions in neurons. The knockdown of *gtr-1* (I) modulates the expression of the neuropeptide-coding genes, *nlp-29*, *nlp-34*, *nlp-56*, and *nlp-59* (II). These modulations are associated with the activation of SKN-1 in Aβ-expressing cells that changes the worm’s transcriptomic landscape (IV), reduces the rate of Aβ aggregation, and enhances protein degradation (V), to mitigate Aβ-mediated proteotoxicity (VI). The same modulation of neuropeptide-coding genes (II) is associated with the activation of DAF-16 in poly35-YFP–expressing worms to modify gene expression (VIII), enhance polyQ35-YFP aggregation, and elevate protein degradation (IX). These effects alleviate the toxicity of polyQ35-YFP (X).

To differentially respond to dissimilar challenges, neurons must know which proteotoxic protein challenges remote cells. However, it is unclear how this information flows from distal tissues to the neuronal system. One possible explanation suggests that muscle cells, which express both proteotoxic proteins in the worm models that were used here, directly inform neurons what insult challenges them, possibly through glial cells. This assumption relies on the description of a muscle-to-glia signaling (*47*) and on the observation that glial cells secret neuropeptides that coordinate proteostasis across tissues (*34*). An alternative possibility proposes a more complex mechanism which involves additional tissues to transmit this information. Such mechanism may involve the gut, a tissue that appears to be a proteostasis signaling tissue (*48*), which also regulates fat metabolism via signaling to the nervous system (*49*). The high expression levels of *nlp-29* and *nlp-34* in the hypodermis (*33*) suggest that muscle cells may communicate with hypodermal cells to indirectly inform neurons what aggregative protein challenges their proteostasis. The opposing effects of *gtr-1* RNAi on the expression levels of *nlp-39* and *nlp-59* in Aβ- and polyQ35-YFP–expressing worms may support this theme. Yet, key mechanistic questions regarding the involvement of neuropeptides in the orchestration of proteostasis across the organism remain to be addressed. Whether these neuropeptides are released from *gtr-1* expressing neurons, from other neurons, or from non-neuronal tissues and what mechanism promotes their release upon the knockdown of *gtr-1* are among these unanswered questions. The elucidation of these fascinating queries requires additional research.

The observation that the knockdown of *gtr-1* needs two different transcription factors, SKN-1 and DAF-16, to protect worms from Aβ and polyQ35-YFP is also puzzling. First, DAF-16 is required for the mitigation of Aβ-mediated proteotoxicity upon the knockdown of *daf-2* (*11*). Therefore, it is likely that the knockdown of *gtr-1* and *daf-2* activate distinct counter-proteotoxic mechanisms to reduce Aβ toxicity and that these alternative mechanisms require different transcription factors to mitigate proteotoxicity. This hypothesis is consistent with the observation that while the knockdown of *daf-2* promotes Aβ hyperaggregation (*11*), *gtr-1* RNAi treatment leads to disaggregation (Fig. 1G). Second, it suggests that neurons specifically activate transcription factors in distal tissues according to the proteotoxic insult that challenges these remote cells. Differential control of transcription factors in somatic cells by neurons has been already reported (*50*). Nevertheless, a set of experiments using *daf-16* and *skn-1* mutants, as well as tissue-specific rescue, will be needed to further substantiate the roles of these transcription factors in the promotion of proteostasis by the knockdown of *gtr-1* and to define their tissues of activity.

Another puzzling aspect of this study is the relations between GTR-1 and DAF-16. On one hand, DAF-16 is needed for the protection from polyQ35-YFP (Fig. 2E), but not from Aβ (Fig. 2B), that is mediated by *gtr-1* RNAi. In addition, the knockdown of *gtr-1* and *daf-16* by RNAi similarly affects the expression levels of known DAF-16 target genes (fig. S4, G and H). However, on the other hand, the knockdown of *gtr-1* results in reduction of DAF-16::GFP (fig. S6, D and E). One possible explanation to this apparent discrepancy suggests that the knockdown of *gtr-1* dissimilarly affects the expression levels of *daf-16* in the face of different proteotoxic challenges. An alternative theme proposes that a residual DAF-16 activity in certain tissues may respond to proteotoxicity despite the reduction in overall DAF-16 levels. To understand the relations between these two key proteins, DAF-16 and GTR-1, it is needed to map the rates of DAF-16 activities in different tissues of *gtr-1* RNAi–treated worms.

In this regard, it is intriguing that while the knockdown of *gtr-1* and of a nucleolar proteostasis-promoting mechanism (*51*) enhances UPS activity, the inhibition of IGF1 signaling in mammalian cells reduces this activity (*52*). This further implies that at least two distinct mechanisms can be activated to alleviate the toxicity of Aβ under different circumstances. Together, these observations culminate to show that neurons not only activate distinct proteostasis-promoting mechanisms to enable remote cells coping with different proteotoxic challenges but also can regulate the activities of dissimilar mechanisms to reduce the toxicity of the same aggregative protein in distal tissues. These differential activation modes are achieved by the secretion of distinct messengers. We previously characterized a set of neuropeptides whose expression levels are elevated upon the knockdown of *torsinA*, a treatment that ameliorates Aβ-mediated toxicity (*24*). Unexpectedly, the neuropeptides that show modified expression levels upon the knockdown of *gtr-1* and are involved in countering Aβ proteotoxicity do not overlap with those required for reducing Aβ toxicity upon the knockdown of *torsinA.* This further suggests that distinct counter-Aβ mechanisms are activated upon distinct proteostasis-promoting RNAi treatments.

This study highlights the importance of elucidating proteostasis-enhancing neuronal signaling mechanisms, as this knowledge may set the basis for the development of future disease-specific preventive remedies for neurodegenerative illnesses. In this context, it is important to note that fundamental proteostasis mechanisms have been shown to be conserved from worms to mammals. For instance, the roles of the IIS as a modulator of proteostasis, which were first found in *C. elegans* (*11*), have been shown to be conserved in mice (*15*, *16*, *53*). Likewise, the neurotransmitter serotonin was reported to regulate proteostasis in worms (*54*) and later to mitigate the toxicity of ataxin-3 in mice (*55*). Last, a screen that was conducted in *C. elegans* pointed at a therapeutic compound that later was shown to mitigate the symptoms of amyotrophic lateral sclerosis in humans (*56*). Together, these studies emphasize the importance of nematodes in basic and clinically related research.

## MATERIALS AND METHODS

### *Caenorhabditis elegans* strains and growth conditions

All worm strains (listed in table S1) were grown on standard nematode growth media (NGM) plates, at 20°C (unless indicated otherwise) and fed with *Escherichia coli* HT115 bacteria. Worm strains used in this paper are listed in table S1. CF512 worms are heat sensitive feminized and thus were routinely incubated at 15°C. To avoid progeny in experiments, synchronized eggs of CF512 worms were incubated at 25°C for 72 hours and transferred back to 20°C until harvested (no ethical approval is needed for the conduction of worm-based experiments).

### RNA interference

All RNAi (listed in tables S2 and S3) treatments were applied using NGM-ampicillin plates seeded with HT115 *E. coli* bacteria harboring the indicated RNAi plasmid. The bacteria were grown overnight at 37°C in Luria-Bertani broth medium containing ampicillin (100 μg/ml). Before spotting on plates, the bacterial cultures were supplemented with 100 mM isopropyl-β-d-thiogalactopyranoside (IPTG; ∼4 mM final concentration) to induce the expression of dsRNA. All RNAi strains used in this study and their sources are listed in table S2. An empty RNAi L4440 plasmid (EV) was used as a control.

### Paralysis assays

Synchronized CL2006 eggs were placed on NGM-ampicillin plates seeded with the desired bacteria and supplemented with IPTG (∼4 mM final concentration). The worms were allowed to complete development. On day 2 of adulthood, 130 animals were transferred onto small NGM plates seeded with the respective *E. coli* culture. Rates of paralysis were recorded daily by tapping the worm noses with a platinum wire. A paralyzed animal was defined as an animal that is able to move its head but unable to crawl away. The assays were terminated at day 12 of adulthood, when wild-type animals start exhibiting age-related paralysis.

### Thrashing assays

Thrashing rate was determined by transferring an individual animal into a drop of 10-μl M9 buffer at day 4 or 6 of adulthood, allowing 30 s of adaptation and counting the number of body bends during the following 30 s. A body bend was defined as the change in direction of the bend in the mid-body of an animal. Twenty animals per treatment in each independent experiment were used, and at least two independent experiments were performed.

### Native agarose gel electrophoresis

NAGE experiments were conducted according to the protocol of Holmberg and Nollen. In brief, 12,000 AM140 eggs were placed on NGM plates seeded with bacteria that express either EV or *gtr-1* 3′ untranslated region (3′UTR) RNAi. The worms were developed at 20°C and washed daily from day 1 of adulthood to get rid of progeny. Half of the worms of each treatment were collected on day 4, while the other half were collected on day 6. The worms were washed with phosphate-buffered saline (PBS), supplemented with protease inhibitor cocktail (Rosch), snap frozen in liquid nitrogen, and stored in −80°C. Frozen samples were thawed on ice, and the worms were homogenized using a bullet blender. Homogenates were spun at 1000*g* for 4 min in 4°C, supernatants were transferred to new tubes, and protein concentrations were determined by a bicinchoninic acid (BCA) kit (Pierce no. 23227, Thermo Fisher Scientific, Waltham, MA, USA). Protein samples (30 μg) were supplemented (1:5) with NAGE loading buffer [62.5 mM tris-HCl, 10% (w/v) glycerol, and bromophenol blue (pH = 8.7)] and run in an 1% (w/v) agarose gel dissolved in NAGE buffer (25 mM tris and 0.19 M glycine with pH = 8.3) and run overnight at 4°C at 50 V. YFP fluorescence was observed using Typhoon FLA 9500.

### Filter-trap assay

AM140 worms were grown on HT115 bacteria harboring the indicated RNAi. On day 4 or 6 of adulthood, the worms were washed with M9 buffer, collected, and frozen in liquid N_2_. The worm pellets were thawed on ice and disrupted using a bullet blender in a lysis buffer [50 mM Hepes (pH 7.4), 150 mM NaCl, 1 mM EDTA, and 1% Triton X-100] supplemented with an EDTA-free protease inhibitor cocktail (Roche). Debris were sediment by low-speed centrifugation (8000*g*, for 5 min, 4°C). One hundred micrograms of protein per sample was supplemented with SDS to the final concentration of 0.5% and loaded onto a cellulose acetate membrane assembled in a slot blot apparatus (Bio-Rad, catalog no.1703938). The membrane was washed with 0.2% SDS, and the retained Q35-YFP was immune-blotted using a GFP antibody (Cell Signaling Technology, catalog no. 2956). For quantification, 100 μg of protein per sample was subjected to WB, and the membranes were blotted with an actin antibody (Sigma-Aldrich, catalog no. A5441).

### Microscopy

Day 1 adult worms were washed twice with M9, anesthetized with 20 mM sodium azide, and mounted on glass slides. Images were taken after clustering worms in the slides using a Nikon AZ100 multizoom microscope. Foci of AM140 were counted and measured using Adobe Photoshop CS4 software, while the intensities of the mCherry and GFP were measured using ImageJ. Exposure time was consistent between treatments of the same set. To assess the rates of cytosolic and nuclear DAF-16, we used worms that express DAF-16 fused to GFP (strain CF1934). The worms were treated with the indicated RNAi bacteria and visualized by fluorescent microscopy at days 1 and 3 of adulthood.

### RNA extraction and next-generation sequencing

Six thousand synchronized CL2006 or AM140 worms were grown on IPTG-induced bacteria expressing either EV or *gtr-1* 3′UTR RNAi. At day 1 of adulthood, the worms were bleached, and eggs were placed on the same RNAi as their parental generation. Worms of the second generation were washed daily to get rid of progeny and harvested at day 6 of adulthood, and RNA was extracted using a kit (Macherey-Nagel no. 740961) according to the manufacturer’s instructions, RNA integrity was tested, and the samples were submitted for NovaSeq RNA-seq. Library preparation for next-generation sequencing: We used an RNA ScreenTape kit (catalog no. 5067-5576; Agilent Technologies), a D1000 ScreenTape kit (catalog no. 5067-5582; Agilent Technologies), a Qubit RNA HS Assay kit (catalog no.Q32852; Invitrogen), and a Qubit DNA HS Assay kit (catalog no. 32854; Invitrogen). mRNA libraries were prepared using a KAPA Stranded mRNA kit with mRNA Capture Beads (KAPA Biosystems, KK8421). In brief, 1 μg was used for the library construction; library was eluted in 20 μl of elution buffer. All DNA sample libraries were pooled into 10 nM samples. Multiplex sample pools were loaded on NovaSeq 6000 (Illumina) using the NovaSeq 6000 SP Reagent Kit v1.5, 100 cycles (catalog no. 20028401), with 122 cycles of single-end sequencing

### Computational analyses of next-generation sequencing data

Raw reads were processed for quality trimming and adaptor removal using fastx_toolkit v0.0.14 and cutadapt v2.10 (*57*). The processed reads were aligned to the *C. elegans* transcriptome and genome version WBcel235 with annotations from Ensembl release 106 using TopHat v2.1.1 (*58*). Counts-per-gene quantification was done with htseq-count v2.01 (*59*). Normalization and differential expression analysis were performed using the DESeq2 package. Genes with a sum of counts less than 10 over all samples were filtered out, and then size factors and dispersion were calculated. Normalized counts were used for several quality control assays, such as counts distributions and principal components analysis, which were calculated and visualized in R. Pairwise comparisons were tested with default parameters (Wald test), except not using the independent filtering algorithm. Significance threshold was taken as padj < 0.01 (default). In addition, genes that exhibited significant changes in expression levels were further filtered by the log2FoldChange value. This filtering was baseMean dependent and required a baseMean above 5 and an absolute log2FoldChange higher than 5/sqrt(baseMean) + 0.6 (for highly expressed genes, this means a requirement for a fold change of at least 1.5, while genes with a very low expression would need a 7-fold change to pass the filtering). While the genes that passed the default (padj < 0.01) filter are termed “Sig,” the subset of genes that also passed the additional filter are termed “Best.” Last, results were combined with gene details (such as symbol, known transcripts, etc.), taken from the results of a BioMart query (Ensembl, release 106), to produce the final Excel file.

#### Gene Ontology classification

All significant gene entries were subjected to KEGG and Gene Ontology (GO) classification (www.geneontology.org). Significant overrepresentation of KEGG- and GO-classified biological processes was assessed by comparing the number of pertinent genes in a given biological process to the total number of the relevant genes printed on the array for that particular biological process (Fisher’s exact test) using the publicly accessible software DAVID (https://davidbioinformatics.nih.gov/). Significant gene entries were also analyzed using the WormCat tool (www.wormcat.com/).

### Identification of genes that opposingly respond to the knockdown of gtr-1 in CL2006 and AM140 worms

To identify genes that respond in opposite to *gtr-1* RNAi in CL2006 versus AM140 worms, we calculated the product of the fold change values of genes differentially expressed in both datasets. Genes whose products resulted in negative values (CL2006 * AM140 < 0) responded in opposition. To visualize these results, the fold change values were plotted on an *xy* axis, and values plotted on the second and fourth quadrants were annotated and marked in red.

### RNA isolation and qPCR

Total RNA was isolated using a NucleoSpin RNA isolation kit (Macherey-Nagel, no. 740955). For each time point, 10,000 synchronized eggs were placed on NGM-ampicillin plates seeded with the indicated RNAi bacteria and supplemented with IPTG. The worms were washed daily with M9 to get rid of progeny, and adult nematodes were transferred to new plates. At the indicated age, the worms were harvested in M9 and frozen at −80°C. The worms were then thawed and homogenized using a Dounce homogenizer. Homogenates were transferred to Eppendorf tubes and centrifuged at 14,000*g* for 5 min. The supernatants were transferred to NucleoSpin Filter (NucleoSpin RNA kit, Macherey-Nagel, Düren, Germany), and total RNA was purified according to the manufacturer’s instructions. The RNA was quantified using a NanoDrop 2000c spectrophotometer.

For qPCR: cDNA was prepared by reverse transcription of the total RNA samples via random-priming using the iScript cDNA Synthesis Kit (no. 170-8890; Bio-Rad, Hercules, CA, USA) as per the manufacturer’s protocol. Analyses by qPCR were performed using gene-specific primers (table S4). Analyses by qPCR were performed with Luna Universal qPCR Master Mix (Biolabs, catalog no. M3003G), and expression levels were normalized to the expression levels of *cdc-42* and *pmp-3*, which served as normalizing genes. qPCRs for each gene were performed in triplicate.

### Identification of daf-16 targets in gtr-1 RNAi–treated proteotoxicity models

DEGs were identified from RNA-seq data generated in two *C. elegans* proteotoxicity models, AM140 (polyQ35) and CL2006 (Aβ), following gtr-1 RNAi compared to EV control. DAF-16 transcriptional targets were defined using the consensus ranking from Tepper *et al.* (*38*).

To visualize the shared transcriptional response between models, genes differentially expressed in both AM140 and CL2006 were identified by intersecting the two DEG sets. Log_2_ fold changes for these shared DEGs were plotted against each other in a scatter plot, with DAF-16 targets highlighted and genes responding in opposing directions between the two models annotated. To characterize model-specific responses, DEGs unique to each strain (present in one dataset but absent from the other) were visualized as volcano plots, with DAF-16 targets colored and the top 20 targets by absolute log_2_ fold change labeled. All visualizations were generated in R using ggplot2, with label placement handled by ggrepel and figure composition by patchwork.

### Protein blotting

For the blotting of Aβ, 12,500 worms were treated from hatching with the indicated RNAi and washed daily from day 1 of adulthood until harvest to get rid of progeny. At day 3, the animals were washed three times with M9, resuspended, supplemented with a protease inhibitor cocktail (Calbiochem, no. 539134), and homogenized using a Dounce B homogenizer. Worm homogenates were spun for 4 min at 850*g* to sediment debris, and supernatants were collected. Protein concentrations were measured by a BCA kit (Pierce, no. 23227, Thermo Fisher Scientific, Waltham, MA, USA). Each soluble protein sample (50 μg) was supplemented with X4 loading buffer [40% glycerol, 125 mM tris (pH = 6.8), 4% SDS, 0.5% bromophenol blue, and 12% β-mercaptoethanol] and heated to 95°C for 5 min. Worm debris were resuspended in M9, supplemented with loading buffer, and heated as above. Supernatants and debris were separated on 12% polyacrylamide gel, then transferred onto a polyvinylidene difluoride membrane, and blotted with anti-Aβ antibody (clone 6E10, BioLegend no. 803001). For the blotting of GFP and ubiquitin, only supernatants were loaded onto the gels. A GFP antibody (Cell Signaling Technology, no. 2956) or the anti-FK2 like ubiquitin antibody (UBCJ2, Enzo, ENZ-ABS840) was used (antibodies are listed in table S5). A chemiluminescence system (Chemidoc XRS+; Bio-Rad) was used to detect protein signals. For signal quantification purposes, the membranes were stripped and reblotted with an anti–β-actin primary antibody (Sigma-Aldrich, no. A1978).

### GB123-based cathepsin B labeling assay

Twelve thousand synchronized eggs of CF512 worms were placed on NGM plates seeded with either EV or *gtr-1* 3′UTR RNAi bacteria. The plates were incubated at 25°C for 72 hours to feminize the worms and transferred to 20°C thereafter. At day 3 of adulthood, the animals were washed three times with PBS and homogenized using a Dounce homogenizer. Homogenates were centrifuged at 14,000*g* at 4°C for 10 min, supernatants were transferred into new tubes, and protein concentrations were determined using a BCA kit. Equal amounts of proteins were incubated at 37°C with GB123 for 2 hours in reaction buffer [50 mM acetate, 4 mM dithiothreitol (DTT), and 5 mM MgCl_2_ (pH 5.5)]. The reaction was stopped by the addition of sample buffer x 4 [40% glycerol, 0.2 M tris-HCl (pH 6.8), 20% β-mercaptoethanol, 12% SDS, and bromophenol blue (0.4 mg/ml)] and boiled for 10 min. Samples were separated by a 12.5% SDS-PAGE, and scanned for fluorescence using a Typhoon scanner FLA 9500 at excitation/emission wavelengths of 633/670 nm.

### In-vitro chymotrypsin-like proteasome activity assay

Twelve thousand synchronized eggs of CF512 worms per treatment were placed on NGM plates seeded with either EV or RNAi-expressing bacteria. The plates were incubated at 25°C for 72 hours to feminize the worms and transferred to 20°C thereafter. On day 3 of adulthood, the worms were washed with PBS three times and homogenized using a Dounce homogenizer. Homogenates were centrifuged at 10,000*g* at 4°C for 15 min, protein concentrations were determined using a BCA kit, and 20 μg of total protein per sample was loaded into a black 96-well plate together with 2X assay buffer [100 mM tris-Cl (pH 7.5), 2 mM DTT, 10 mM MgCl_2_, 4 mM adenosine triphosphate, and 1 mM EDTA + 0.5 M Sucrose] and PBS + NP-40 (0.5%). The fluorogenic substrate was added: Suc-LLVY-AMC (chymotrypsin-like activity, by Boston Biochem, S-280) to a final concentration of 10 μM. For negative control, 20 μM MG132 was used. Excitation/emission wavelength, 345/445 nm. Measurements were taken every 5 min for 90 min at 37°C using the Infinite M200 Pro (Tecan, Mannedorf, Switzerland) with the Magellan Software. Results were normalized to those at time 0 of each experiment.

### Statistical analysis and software

The results are presented as the means ± SEM of at least three independent, biological repeats of each experiment. Statistical significance of differences was assessed by Student’s *t* test. Both statistical analysis and plotting of the data were performed using GraphPad Prism 7 (GraphPad Software Inc., La Jolla, USA).
