# Supplementary material for: A neuronal GPCR modifies neuropeptide signaling to suppress proteostasis in distal tissues
Source: Sci Adv. 2026 Oct 9;12(41):eady6048. doi: 10.1126/sciadv.ady6048 (PMC13651884; doi:10.1126/sciadv.ady6048)
Supplement: Supplementary file 1 [file sciadv.ady6048_sm.pdf]

Science Advances
AAAS

Supplementary Materials for

A neuronal GPCR modifies neuropeptide signaling to suppress proteostasis in distal tissues

Joud Hirbawi *et al.*

Corresponding author: Ehud Cohen, ehudc@ekmd.huji.ac.il

Sci. Adv. **12**, eady6048 (2026)
DOI: 10.1126/sciadv.ady6048

This PDF file includes:

Figs. S1 to S6
Tables S1 to S5

Supplemental figure 1

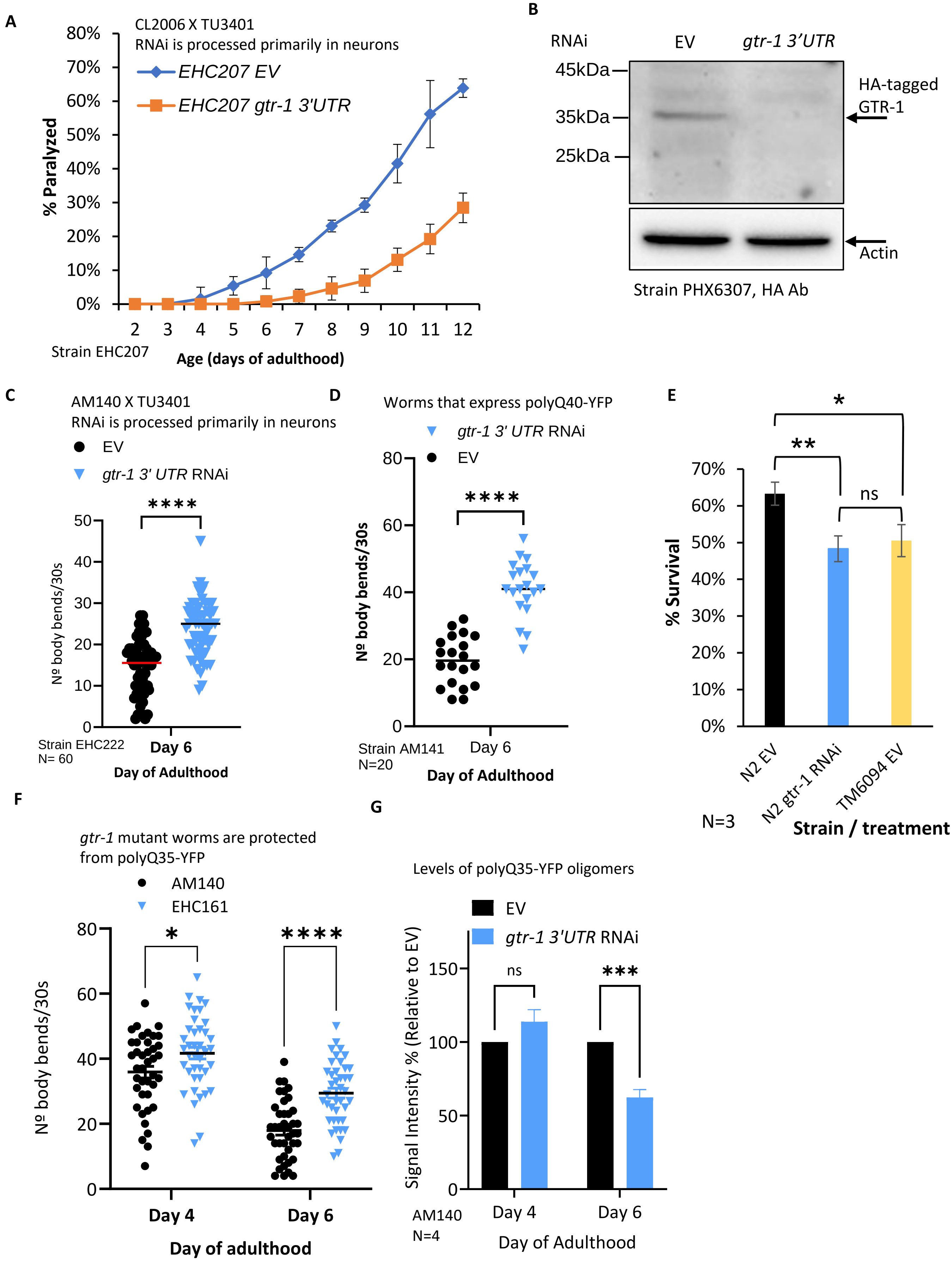

Supplemental Figure 1

A. EHC207 worms which express Aβ in their muscles and are amenable to RNAi-mediated gene knockdown primarily in neurons, are largely protected from the toxicity of Aβ when treated with *gtr-1* RNAi. **B.** The knockdown of *gtr-1* by RNAi greatly reduces the levels of HA-tagged GTR-1. **C.** EHC222 worms that express polyQ35-YFP in their muscles and process RNAi primarily in their neurons, exhibit higher thrashing rates when treated with *gtr-1* RNAi compared to control, untreated animals of the same strain. **D.** The protection that is conferred by *gtr-1* RNAi is not strain specific as *gtr-1* RNAi-treated AM141 nematodes, that express polyQ40-YFP in their muscles, show higher thrashing rates than their untreated counterparts. **E.** *gtr-1* RNAi treated AM140 animals and EHC161 worms which carry a deletion in the sequence of *gtr-1,* exhibit equally reduced survival upon exposure to 35° C for 12 hours. **F**. EHC161 worms exhibit higher thrashing rates at days 4 and 6 of adulthood, compared to AM140 animals that harbor natural *gtr-1*. **G.** Band intensity measurement shows that the knockdown of *gtr-1* by RNAi reduces the levels of polyQ35-YFP oligomers as compared by native agarose gels (NAGE)**.** (*p<0.05, **p<0.01, ***p<0.001, ****p<0.0001 significance in Fig S1, C and D was calculated using paired T test, in Fig. S1E significance was calculated using one-way ANOVA test and in Fig. S1, F and G using two-way ANOVA test).

Supplemental figure 2

A

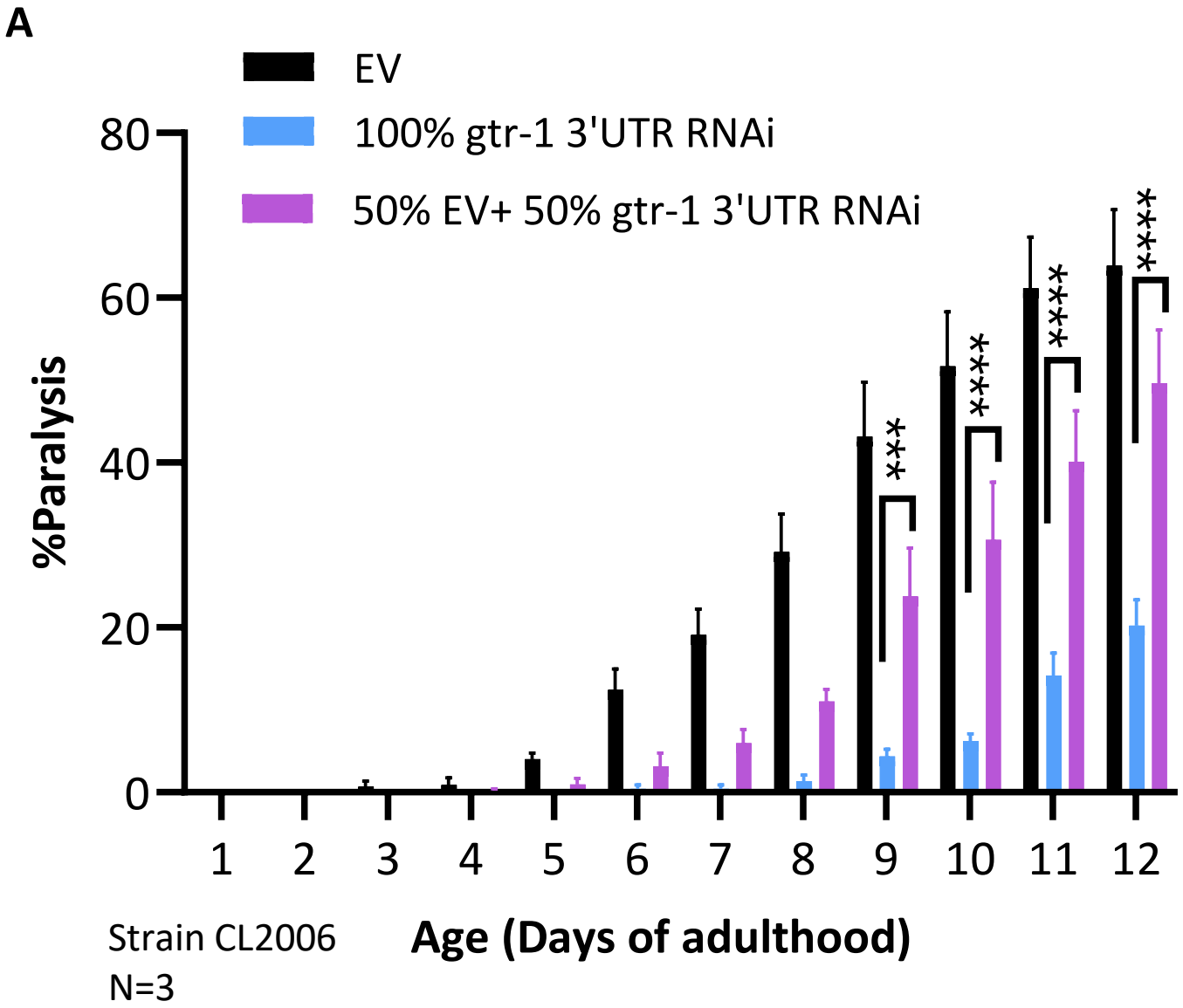

B

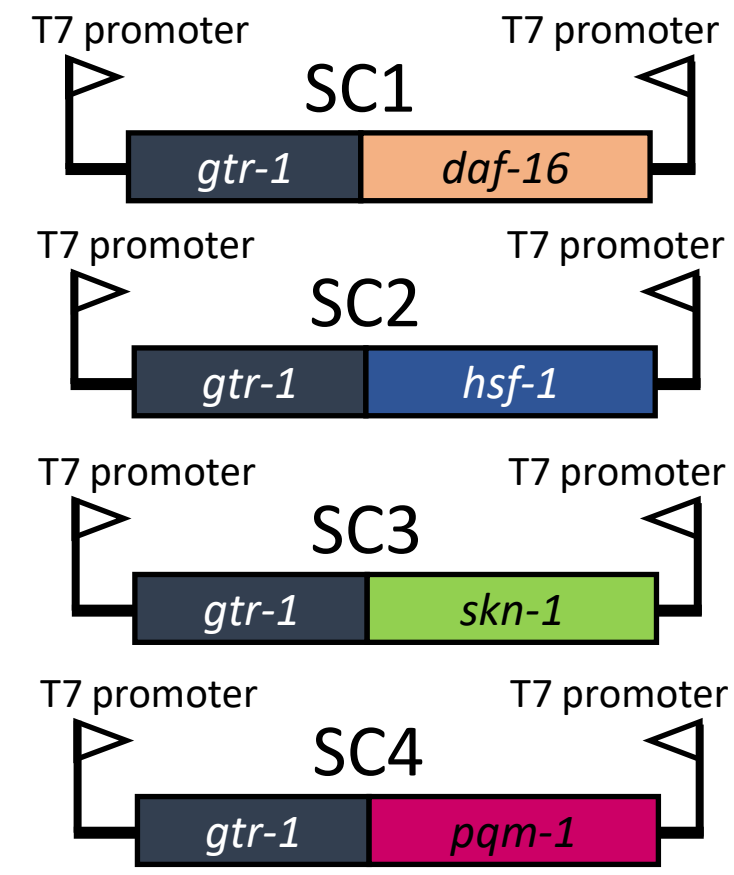

C

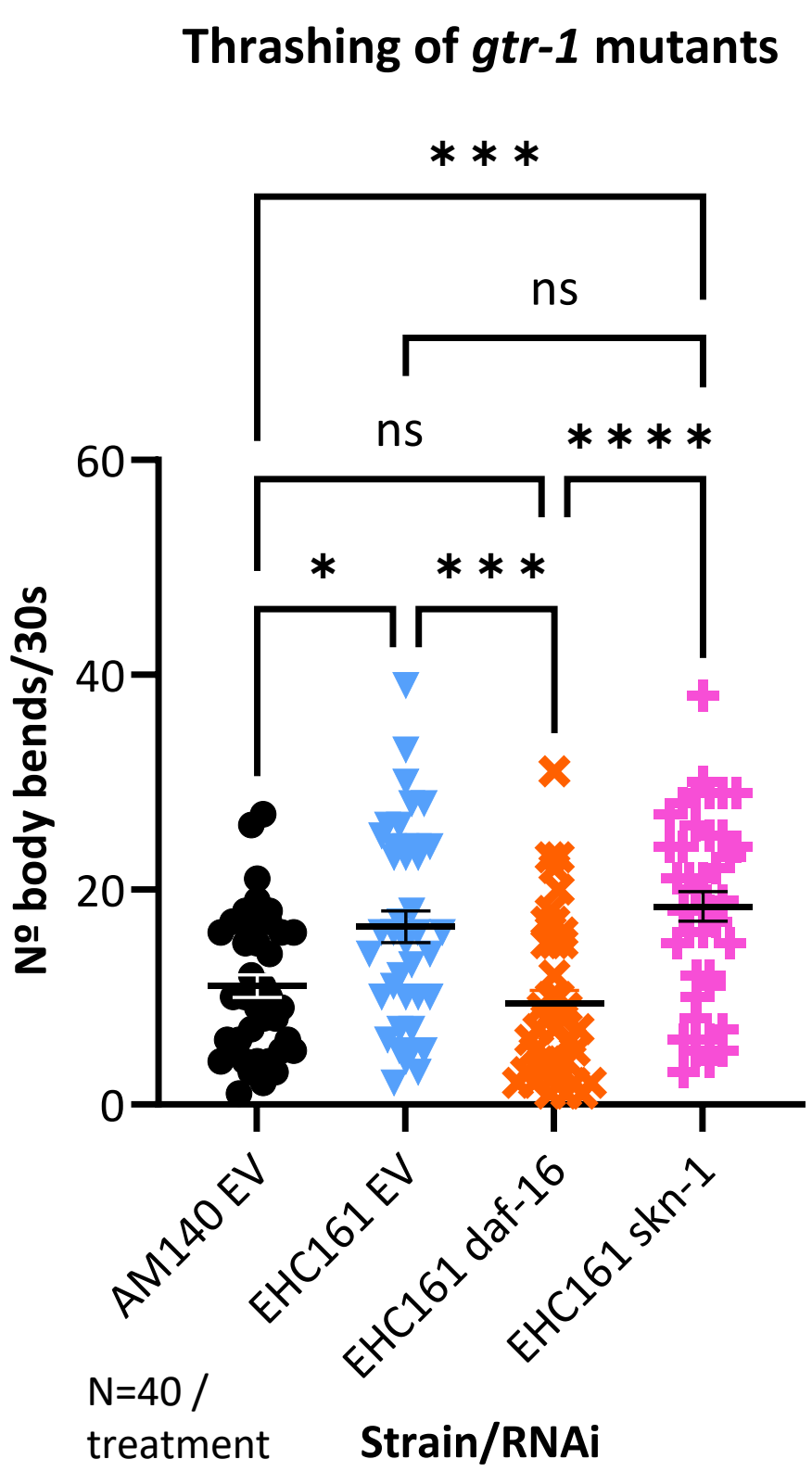

Supplemental Figure 2

A. Paralysis assays using CL2006 worms show that the dilution of *gtr-1* RNAi with control bacteria (EV) to 50%, reduces the counter-proteotoxic efficiency of this treatment. **B.** An illustration of dual silencing RNAi cassettes that concurrently knockdown *gtr-1* and one the indicated transcription factor-coding genes. **C.** Thrashing assays using EHC161 worms confirm the requirement of DAF-16, but not of SKN-1, for the *gtr-1* RNAi-mediated protection from polyQ35-YFP toxicity. (*p<0.05, **p<0.01, ***p<0.001, ****p<0.0001 significance in Fig. S2A was calculated using two-way ANOVA test, and in Fig. S2C one-way ANOVA test).

Supplemental figure 3

A

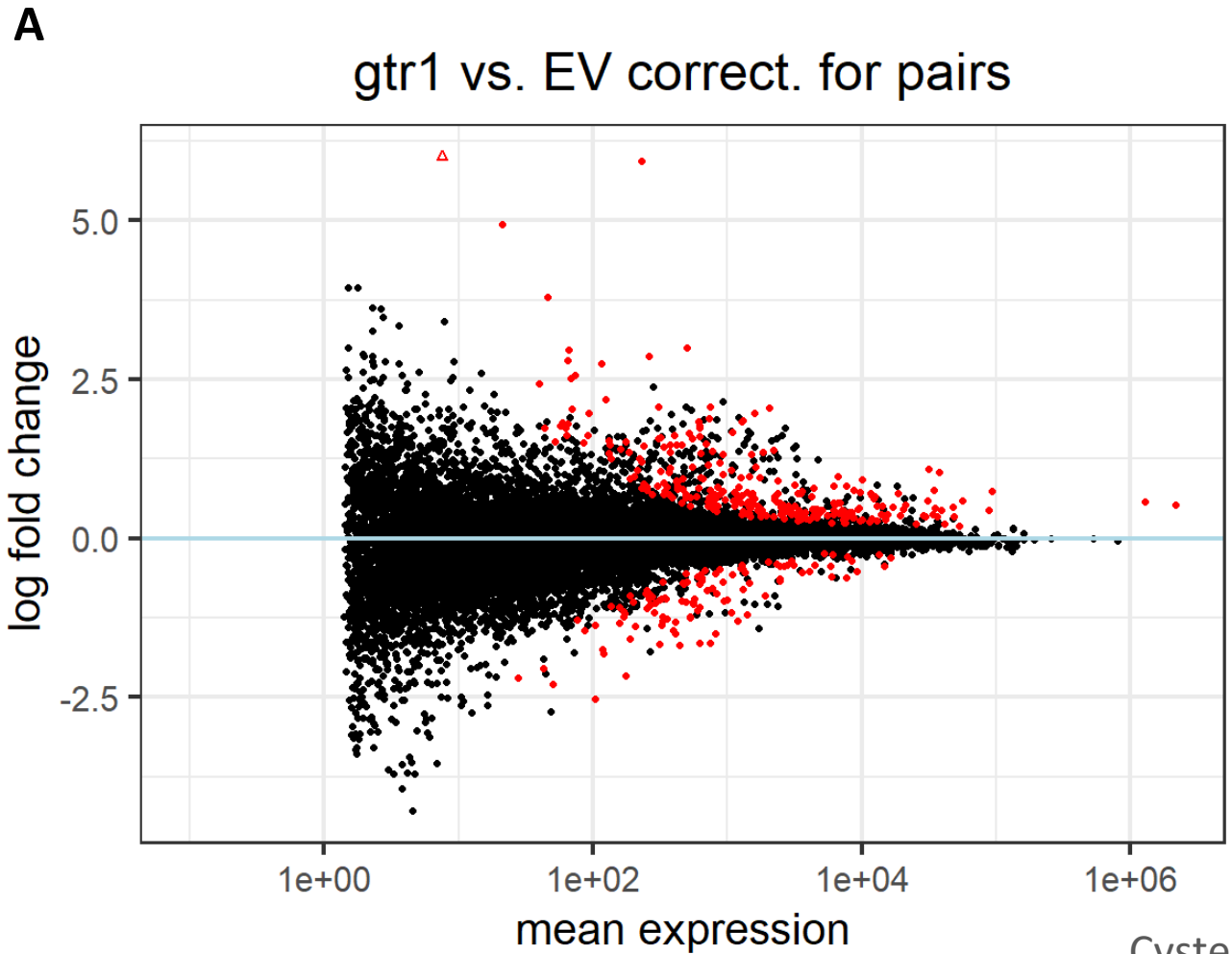

Strain CL2006

B

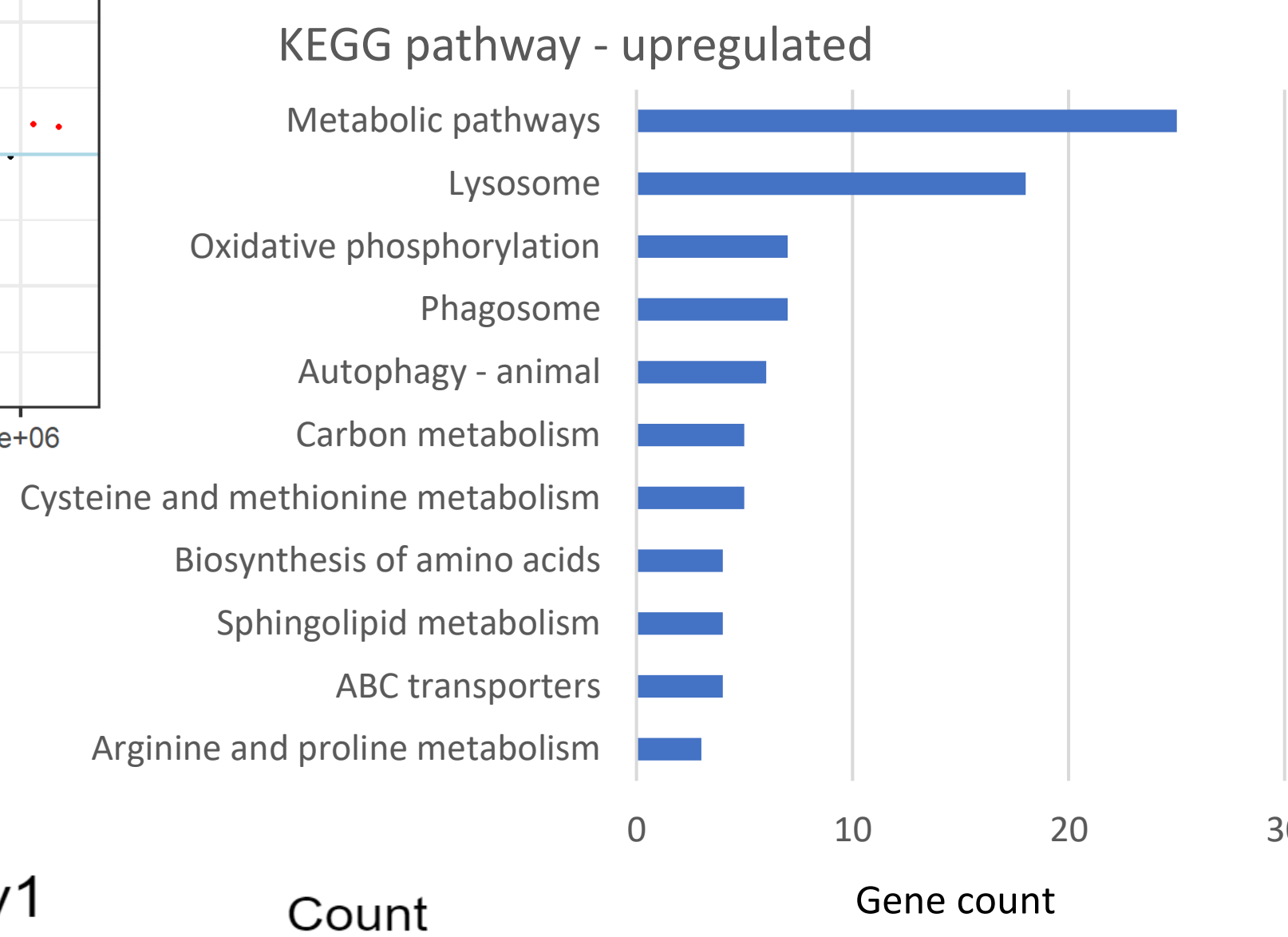

C

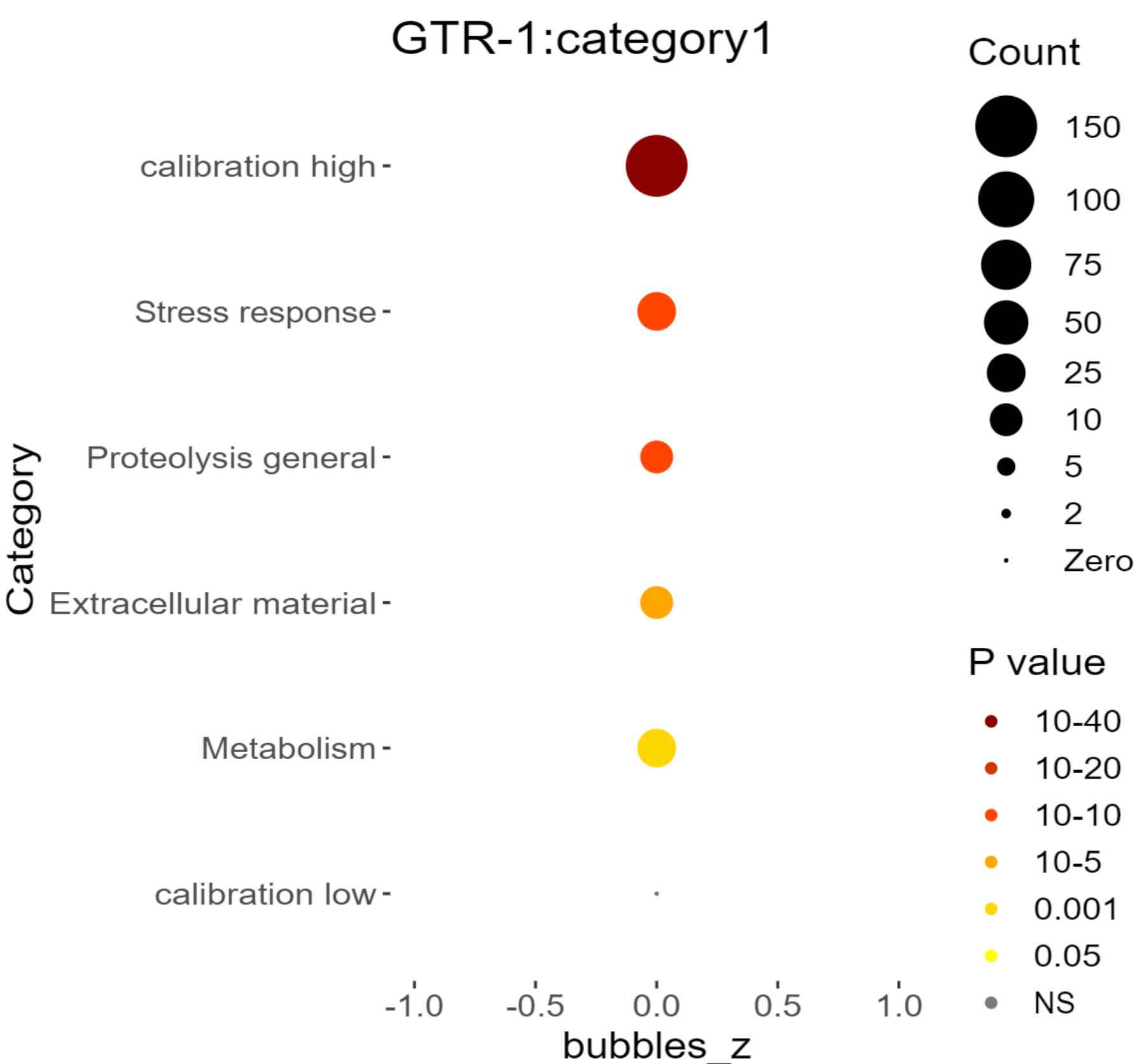

Supplemental Figure 3

A. A volcano plot shows genes that exhibit modulated expression levels upon the knockdown of *gtr-1* by RNAi in CL2006 animals. **B.** KEGG pathway analysis indicates an enrichment of genes that are involved in metabolism and lysosomal components among the genes that exhibit modulated expression levels in *gtr-1* RNAi-treated CL2006 worms. **C.** Gene clustering using the WormCat tool, highlights an enrichment of genes that are involved in stress response and proteolysis among the genes that their expression levels were modulated upon the knockdown of *gtr-1*.

Supplemental figure 4

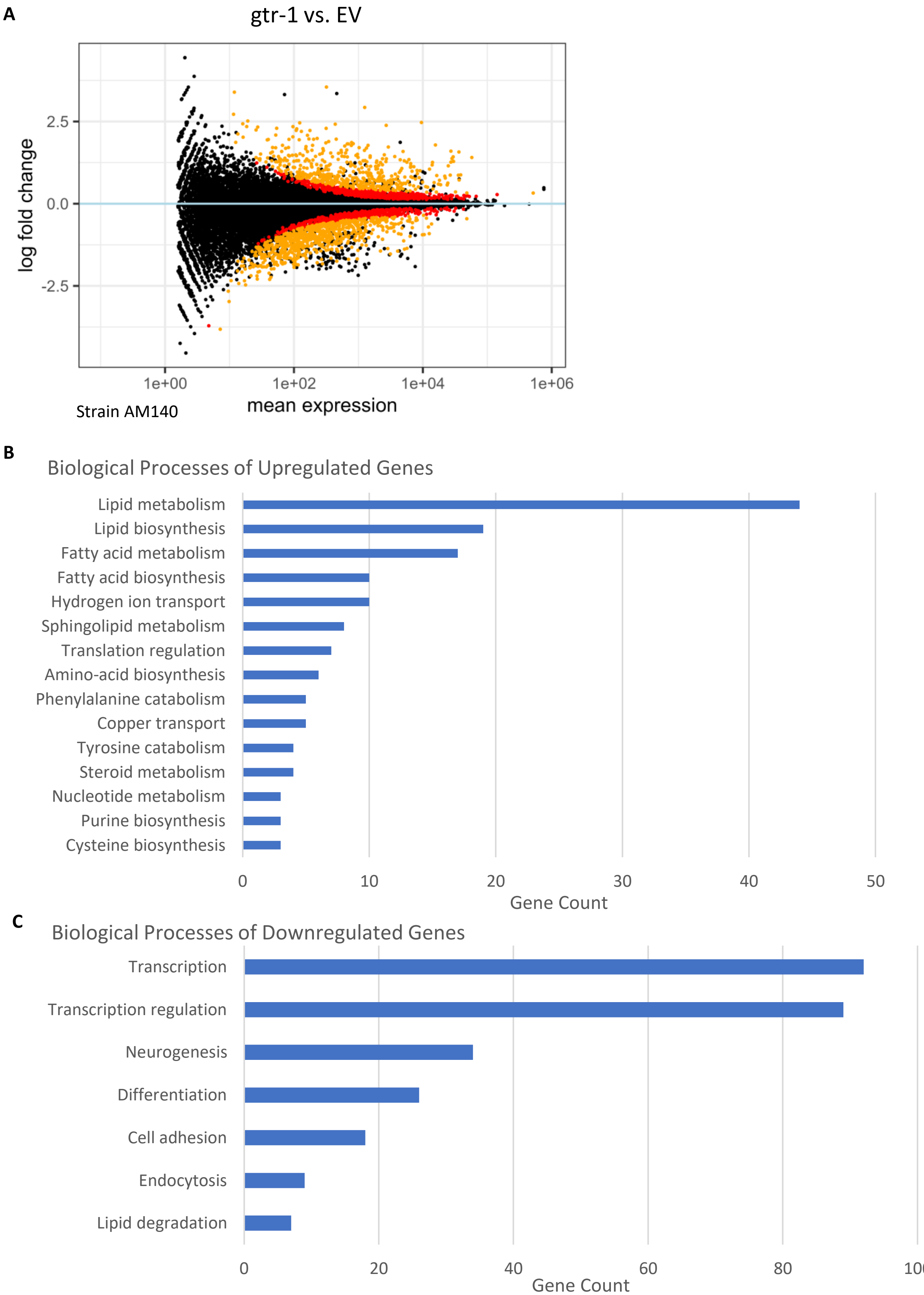

Supplemental figure 4

D

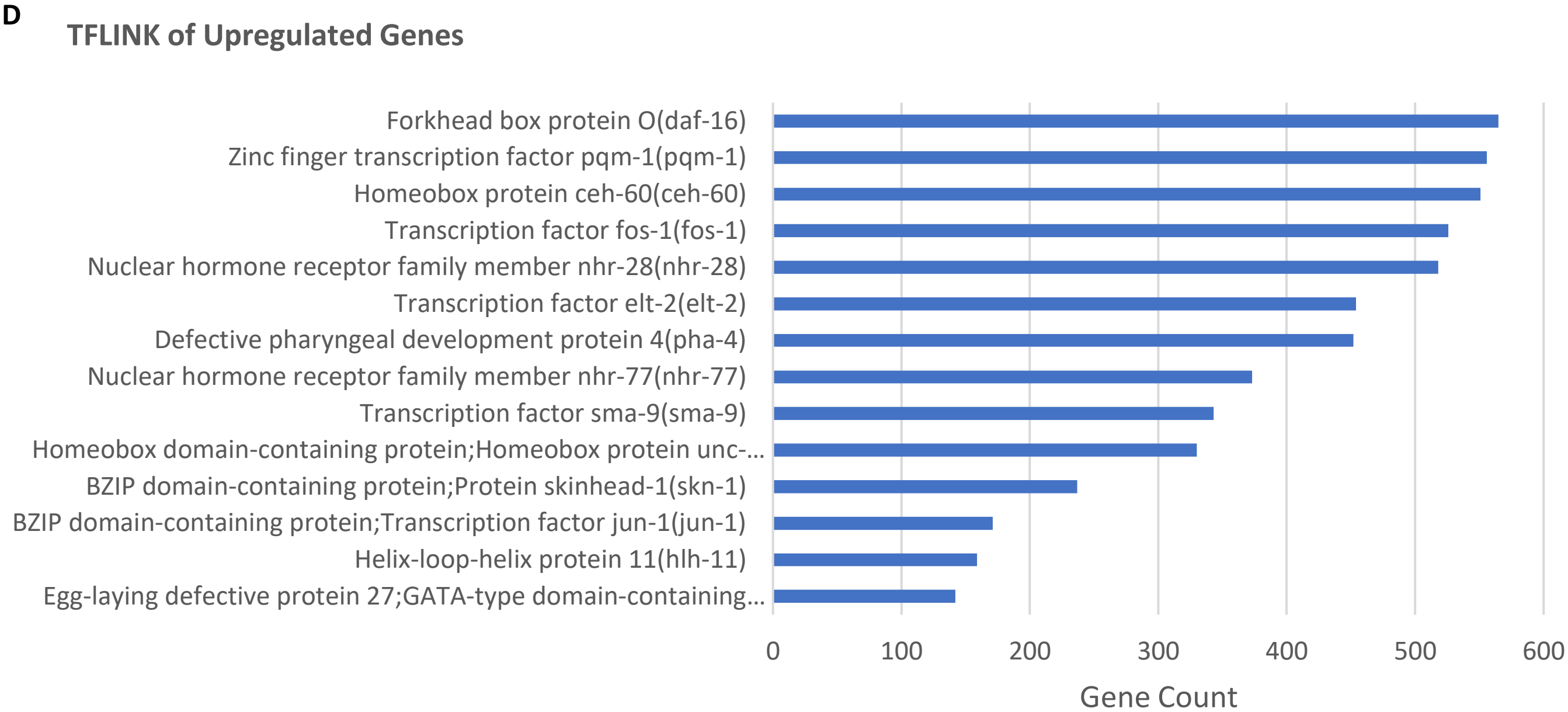

E

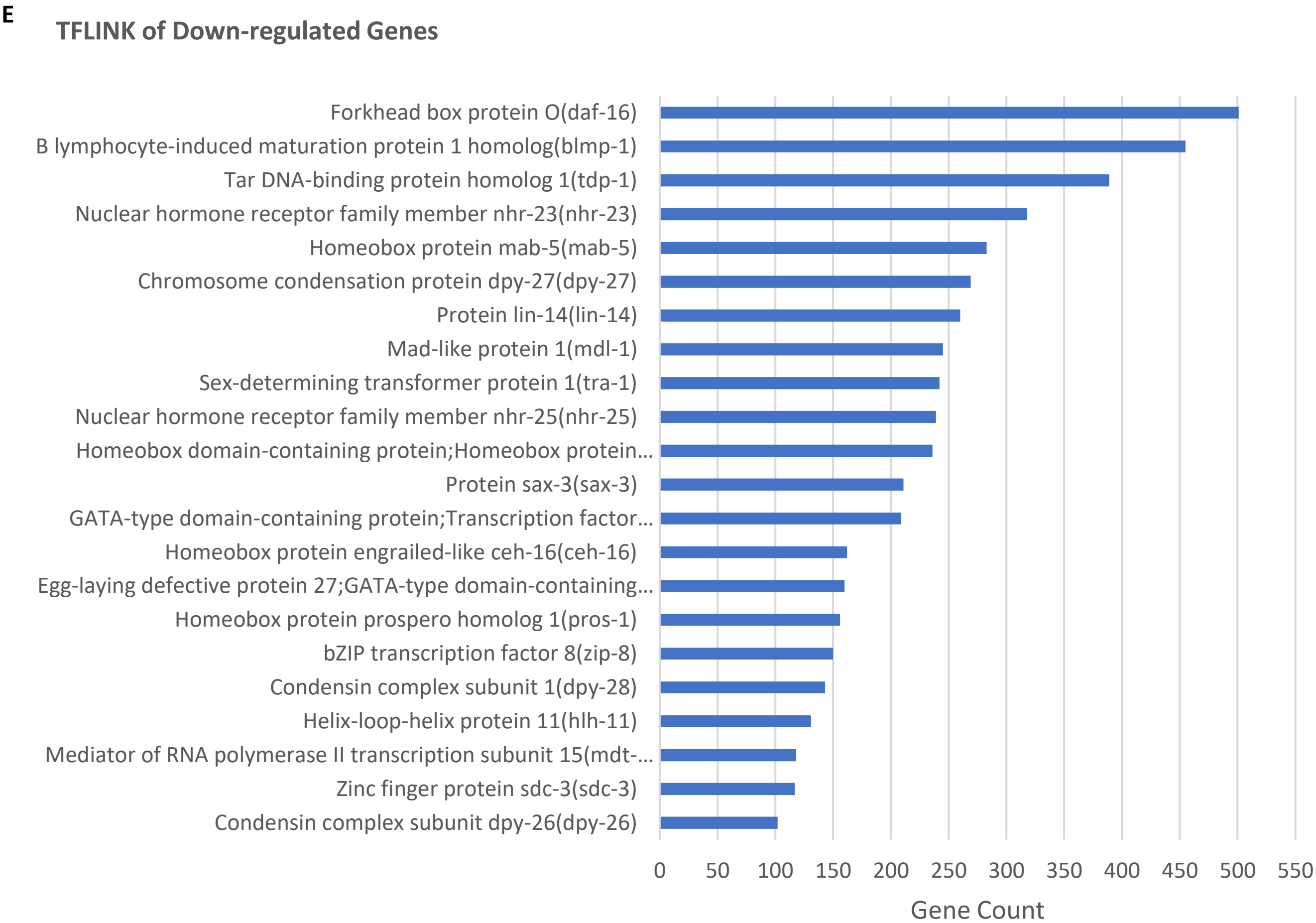

Supplemental figure 4

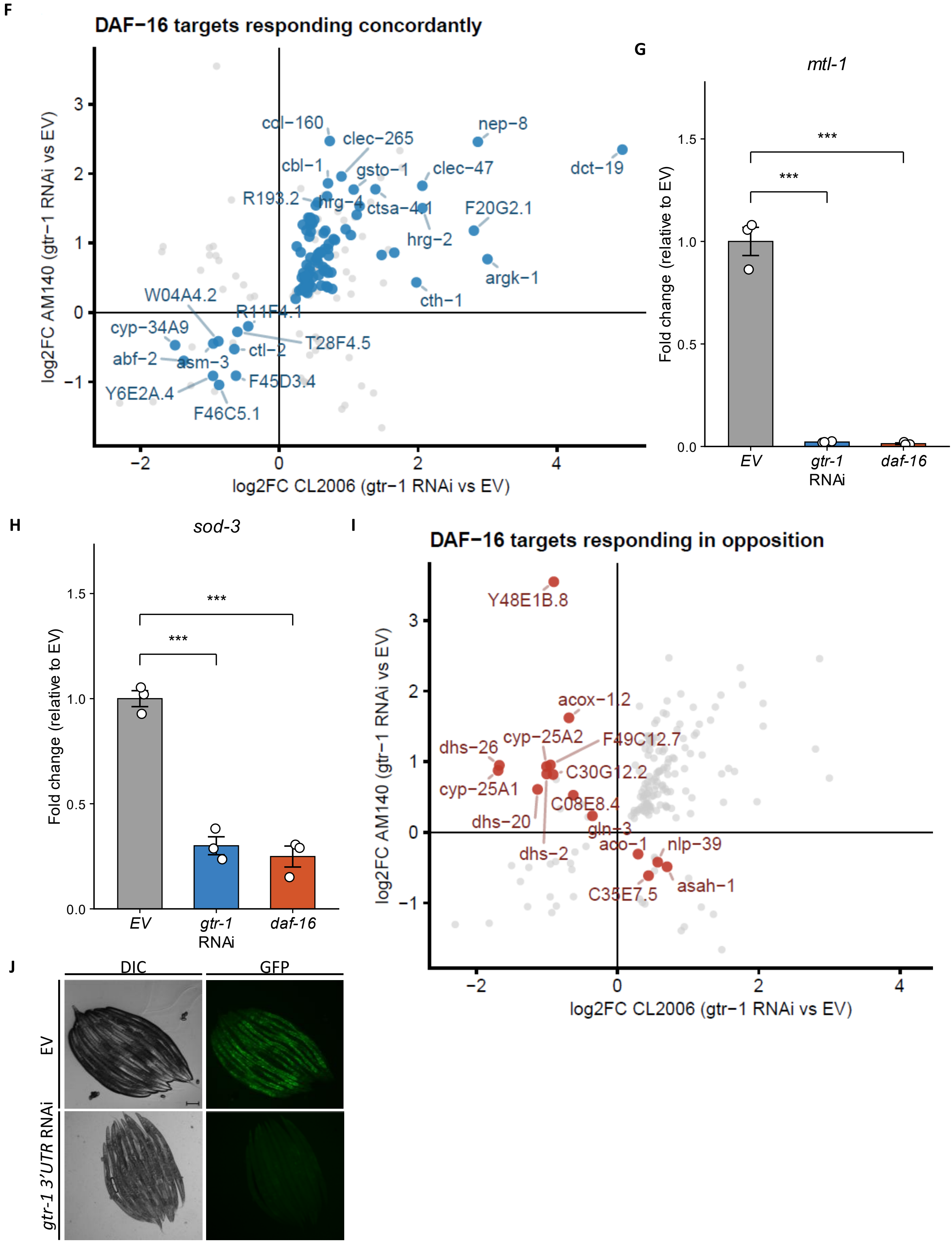

Strain CF1934, Day 1

Supplemental Figure 4

A. A volcano plot shows genes that exhibit modulated expression levels (up and downregulated) upon the knockdown of *gtr-1* by RNAi in AM140 worms. **B.** KEGG pathway analysis of upregulated genes, shows an enrichment of genes that are involved in lipid metabolism and biosynthesis among the genes that exhibit modulated expression levels in *gtr-1* RNAi-treated AM!40 worms. **C.** Analysis of significantly downregulated genes by KEGG pathway analysis shows an enrichment of genes that are involved in transcription and transcription regulation in *gtr-1* RNAi-treated AM140 animals. **D-E.** Clustering the genes that exhibit significantly modulated expression levels upon the knockdown of *gtr-1* in AM140 worms, according the transcription that are known to regulate their expression, indicate that DAF-16-controlled genes are most abundant in both, upregulated (D) and downregulated (E) genes. **F.** DAF-16-target genes that respond to the knockdown of *gtr-1* in a concordant manner in AM140 and CL2006 animals. **G-H.** qPCR indicates that two well-characterized DAF-16 targets genes *mtl-1* (G) and *sod-3* (H) exhibit similarly reduced expression levels when the worms were treated with RNAi toward either *gtr-1* or *daf-16.* **I.** Certain DAF-16 target genes respond in opposition to the knockdown of *gtr-1* and of *daf-16* in AM140 and CL2006 animals. **J.** CF1934 worms that were treated from hatching with *gtr-1* RNAi exhibit reduced GFP signal compared to their untreated counterparts (Scale bar 135µm).

Supplemental figure 5

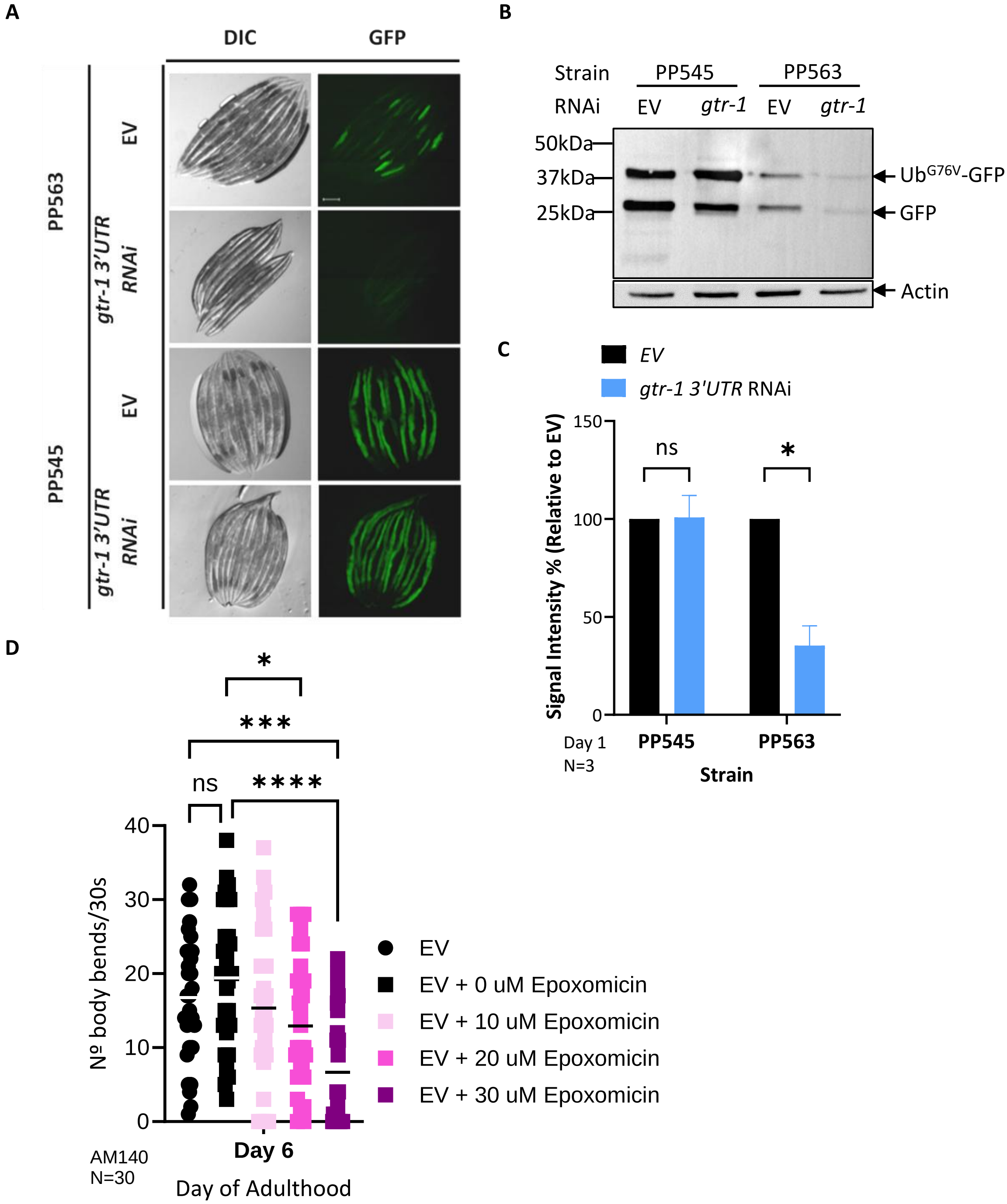

Supplemental Figure 5

The knockdown of *gtr-1* by RNAi enhances proteasome activity. **A-C.** Worms that express the short-lived proteasome substrate Ub^{G76V}-GFP (strain PP563) show lower GFP signal upon treatment with *gtr-1* RNAi, as seen by microscopic visualization (A, scale bar 150µm) and by WB analysis using a GFP antibody (B). Quantification of three independent gels as in (B) confirm the reproducibility and significance of this observation (C). No such effect was seen in PP545 worms which express a stable Ub^{G76V}-GFP protein due to two lysine substitutions in the sequence of ubiquitin in this chimeric protein. **D.** Chemical inhibition of proteasome activity indicates that 30µM Epoxomicin effectively reduce the thrashing rates of 6-day-old AM140 worms. (*$p<0.05$, **$p<0.01$, ***$p<0.001$, ****$p<0.0001$. Significance in Fig. S5C was calculated using multiple T test, in Fig. S5D using one-way ANOVA test).

Supplemental figure 6

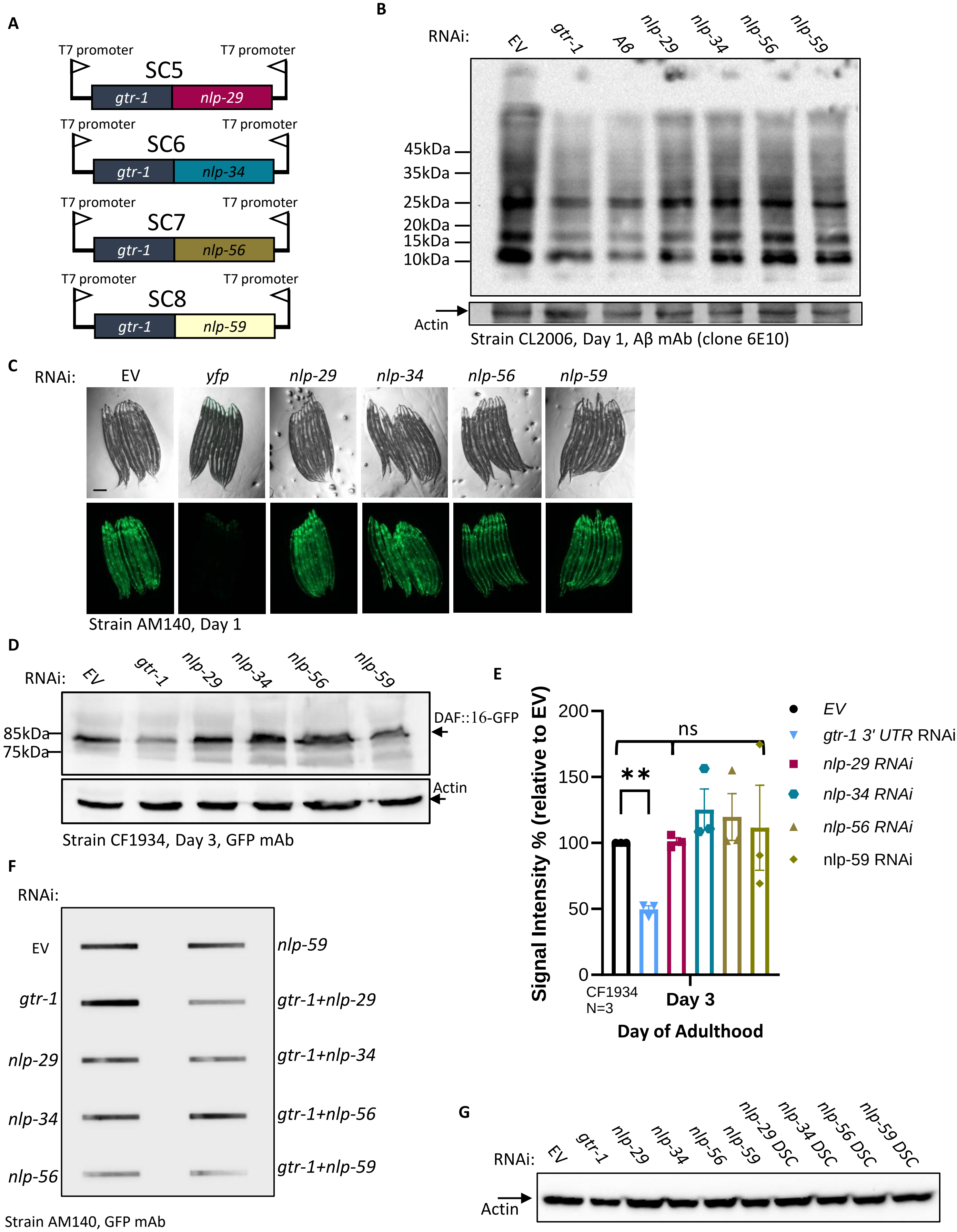

Supplemental Figure 6

A. Illustration of dual-silencing cassettes that were designed to concurrently knockdown the expression of *gtr-1* and one of the indicated neuropeptide-coding genes. **B.** CL2006 worms were grown from hatching on the indicated RNAi bacteria, homogenized at day 1 of adulthood and subjected to WB analysis using an Aβ antibody. While the knockdown of *gtr-1* and of *Aβ* greatly reduced the amounts of the aggregative peptide, the knockdown of either one of the neuropeptides resulted in a moderate reduction in Aβ levels. **C.** AM140 worms were treated from hatching with the indicated RNAi bacteria and visualized by a fluorescent microscope at day 1 of adulthood. The knockdown of neither one of the tested neuropeptide-coding genes significantly affected the YFP signal at this age (Scale bar 150µm). **D-E.** A WB analysis of CF1934 worms that express DAF-16::GFP indicates that the knockdown of *gtr-1* reduces the levels of this chimeric gene (D). No reductions in the levels of DAF-16::GFP were detected when one of the tested neuropeptide-coding genes was knocked down. These effects are reproducible and significant (E). **F.** A filter-trap assay confirms that the knockdown of *gtr-1* by RNAi enhances the aggregation of polyQ35-YFP in AM140 worms. This effect could not be seen when any of the tested neuropeptide-coding genes was knockdown by RNAi, however, a concurrent knockdown of *gtr-1* and of one of the tested neuropeptide-coding genes abolished the aggregation of polyQ35-YFP. **G.** Loading control for E (*$p<0.05$, **$p<0.01$, ***$p<0.001$, ****$p<0.0001$. Significance in Fig. S6D was calculated using one-way ANOVA test).

Table S1: worm strains

Strain	Genotype	Source
N2	Wild-type Bristol	CGC
CL2006	pCL12 (unc-54/human Aβ peptide 1-42 minigene) + pRF4	CGC
PP563	unc-119(ed4); hhIs64[unc-119(+); sur-5::UbV-GFP]III	From Dr. Thorsten Hoppe
PP545	unc-119(ed4); hhIs53[unc-119(+); sur-5::K29/48R-UbG76V-GFP	From Dr. Thorsten Hoppe
EHC207	CL2006 X TU3401	This study: neuron-specific RNAi and Aβ in muscle
AM140	rmIs132 [unc-54p::Q35::YFP]	CGC
MAH215	sqIs11 [lgg-1p::mCherry::GFP::lgg-1 + rol-6].	CGC
CF512	fer-15(b26) II;fem-1(hc17)IV	CGC
PHX6307	3xHA-gtr-1 (by CRISPR technology)	Sunny Biotech China
tm6094	308 bp deletion in F25E2.1- coding for gtr-1	NBRP (the Japanese C. elegans consurtium)
EHC161	AM140 X tm6094	This study: deletion in gtr-1 and polyQ35-YFP in muscle
CF1934	daf-16p::DAF-16::GFP on the background of daf-16 mutants (mu86)	From Dr. Andrew Dillin
EHC222	AM140 X TU3401	This study: neuron-specific RNAi and polyQ35-YFP in muscle
AM141	rmIs132 [unc-54p::Q40::YFP]	CGC

Table S2 – RNAi

**RNAi**	**Source**	**Description**
EV	Dr. Andrew Dillin	
daf-2	Dr. Andrew Dillin	
gtr-1	our lab	
gtr-1 3'UTR	our lab	
YFP	our lab	This RNAi targets the RNA of poly Q-YFP stretches
A-beta	Dr. Andrew Dillin	
skn-1	Vidal library	
daf-16	Dr. Andrew Dillin	
hsf-1	Ahringer library	
pqm-1	Vidal library	
DSC: gtr-1+skn-1	this study	simultaneous knockdown of *gtr-1* and *skn-1*
DSC: gtr-1+daf-16	this study	simultaneous knockdown of *gtr-1* and *daf-16*
DSC: gtr-1+hsf-1	this study	simultaneous knockdown of *gtr-1* and *hsf-1*
DSC: gtr-1+pqm-1	this study	simultaneous knockdown of *gtr-1* and *pqm-1*
rpn-6.1	Vidal library	
lgg-1	Vidal library	
nlp-29 3'UTR	this study	RNAi against the 3'UTR of *nlp-29*
nlp-34	this study	RNAi against *nlp-34*
nlp-56	this study	RNAi against *nlp-56*
nlp-59	this study	RNAi against *nlp-59*
DSC: gtr-1+nlp-29	this study	simultaneous knockdown of *gtr-1* and *nlp-29*
DSC: gtr-1+nlp-34	this study	simultaneous knockdown of *gtr-1* and *nlp-34*
DSC: gtr-1+nlp-56	this study	simultaneous knockdown of *gtr-1* and *nlp-56*
DSC: gtr-1+nlp-59	this study	simultaneous knockdown of *gtr-1* and *nlp-59*
DSC:gtr+rpn-6.1	this study	

Table S3 – Primers used for cloning of RNAi plasmids and genotyping:

Gene	Forward	Reverse
gtr-1	Includes KpnI site<br>GAT GGTACC TTGATATCGAATTCCTGC	Includes SalI site.<br>TTTGTCGACGCAGATCTGATATCATC
nlp-29	Includes KpnI site<br>GAT<br>GGTACCGGAATGTATGGAGGATGGGGA	Includes XhoI site<br>GCT CTCGAG<br>TGAGAAATCAGGATATGTACAACGC
nlp-34	Includes KpnI site<br>GAT GGTACC<br>GATTTCCGCCAAGCTCTTCT	Includes HindIII site<br>GCTAAGCTT<br>CCTCCGTATCCTCCGTATCC
nlp-56	Includes KpnI site<br>GAT GGTACC<br>CGTCAGCTAAGGAGTTTCCC	Includes HindIII site<br>GCTAAGCTT<br>TCACTCCTTCTTGGTCTCGT
nlp-59	Includes XhoI site<br>GAT CTCGAG<br>ATGATTGGTGTGGTGTGGAC	Includes HindIII site<br>GCTAAGCTT<br>AAGGGAGATTTCAGGCCAAC

Table S4 - Primers used for measuring mRNA levels by qPCR:

Gene	Forward	Reverse
gtr-1	TATTCTACATGTTTCGGTGG	TAAATGCCACAAAATACGCC
cdc-42	CTGCTGGACAGGAAGATTACG	CTCGGACATTCTCGAATGAAG
pmp-3	GTTCCCGTGTTCATCACTCAT	ACACCGTCGAGAAGCTGTAGA
skn-1	CCA TAT CAC TCA CAG AGA CAC	CCA ACT TTC CGT AGA AAC G
daf-16	CTTCAAGCCAATGCCACTACC	GGAGATGAGTTGGATGTTGATAGC
hsf-1	TTGACGACGACAAGCTTCCAGT	AAAGCTTGCACCAGAATCATCCC
pqm-1	TCCAGAGAGTATCGACCAAGG	TCATTGATATTTGATAAGCCATCTTT
fat-7	TTCTTGTATTCCAGAGAAAGCAC	ATGAATGCTGTCTCCTTCCA
cpr-3	TACGGATGTAAGGGAGGCTA	GGCAGAAGCTCCATAATGC
nep-10	AAATCTCACTCTGGACGCTTC	GAATGGAGCAAGAACAGTCAG
asp-12	TCTAACTTTAAGAATGACGCTGC	AGACACCTCCTCCAACATTC
mtl-1	GAG GCC AGT GAG AAA AAA TGC T	GCT CTG CAC AAT GAC AGT TTG C
sod-3	CTA AGG ATG GTG GAG AAC CTT CA	CGC GCT TAA TAG TGT CCA TCA G

Table S5 – Antibodies

Target protein	Source	Clone	Notes
GFP	Rabbit monoclonal	D5.1	Cell Signalling Cat# 2956
Actin	Mouse monoclonal	AC-15	Sigma Cat# A1978; RRID: AB_476744
Amyloid β	Mouse monoclonal	Clone 6E10	BioLegend Cat #803001; RRID: AB_2564653
HA tag	Mouse monoclonal	16B12	BioLegend Cat #901501
Mono and poly-ubiquitinated proteins	Mouse monoclonal	UBCJ2	ENZO Cat #ENZ-ABS840
